# Mosquito tagging using DNA-barcoded nanoporous protein microcrystals

**DOI:** 10.1093/pnasnexus/pgac190

**Published:** 2022-09-12

**Authors:** Julius D Stuart, Daniel A Hartman, Lyndsey I Gray, Alec A Jones, Natalie R Wickenkamp, Christine Hirt, Aya Safira, April R Regas, Therese M Kondash, Margaret L Yates, Sergei Driga, Christopher D Snow, Rebekah C Kading

**Affiliations:** Department of Chemistry, Colorado State University, Fort Collins, CO 80523, USA; Department of Microbiology, Immunology, and Pathology, Colorado State University, Fort Collins, CO 80523, USA; Department of Entomology, Cornell University, Ithaca, NY 14853, USA; Department of Microbiology, Immunology, and Pathology, Colorado State University, Fort Collins, CO 80523, USA; School of Biomedical Engineering, Colorado State University, Fort Collins, CO 80523, USA; Department of Microbiology, Immunology, and Pathology, Colorado State University, Fort Collins, CO 80523, USA; Department of Microbiology, Immunology, and Pathology, Colorado State University, Fort Collins, CO 80523, USA; School of Biomedical Engineering, Colorado State University, Fort Collins, CO 80523, USA; College of Veterinary Medicine and Biological Sciences, Colorado State University, Fort Collins, CO 80523, USA; Department of Environmental Health and Radiological Sciences, Colorado State University, Fort Collins, CO 80523, USA; H3 Environmental, Albuquerque, NM 87109 (current); Department of Biochemistry and Molecular Biology, Colorado State University, Fort Collins, CO 80523, USA; Department of Chemical and Biological Engineering, Colorado State University, Fort Collins, Colorado 80523, USA; Department of Chemistry, Colorado State University, Fort Collins, CO 80523, USA; School of Biomedical Engineering, Colorado State University, Fort Collins, CO 80523, USA; Department of Biochemistry and Molecular Biology, Colorado State University, Fort Collins, CO 80523, USA; Department of Chemical and Biological Engineering, Colorado State University, Fort Collins, Colorado 80523, USA

**Keywords:** vector biology, mark–release–recapture, mosquito surveillance, porous protein crystals, host-guest crystals

## Abstract

Conventional mosquito marking technology for mark–release–recapture (MRR) is quite limited in terms of information capacity and efficacy. To overcome both challenges, we have engineered, lab-tested, and field-evaluated a new class of marker particles, in which synthetic, short DNA oligonucleotides (DNA barcodes) are adsorbed and protected within tough, crosslinked porous protein microcrystals. Mosquitoes self-mark through ingestion of microcrystals in their larval habitat. Barcoded microcrystals persist trans-stadially through mosquito development if ingested by larvae, do not significantly affect adult mosquito survivorship, and individual barcoded mosquitoes are detectable in pools of up to at least 20 mosquitoes. We have also demonstrated crystal persistence following adult mosquito ingestion. Barcode sequences can be recovered by qPCR and next-generation sequencing (NGS) without detectable amplification of native mosquito DNA. These DNA-laden protein microcrystals have the potential to radically increase the amount of information obtained from future MRR studies compared to previous studies employing conventional mosquito marking materials.

Significance Statement The persistent threat of mosquito-borne disease emergence and transmission necessitates surveillance of infectious mosquito populations over broad geographic regions and an understanding of mosquito dispersal patterns from larval habitats. Here we present the next generation in mosquito marking materials, a system comprised entirely of DNA and protein. This technological innovation will revolutionize the study of arbovirus circulation by enabling mosquito tracking with high spatial resolution and negligible encumbrance to mosquitoes. The result will be new technology for mosquito surveillance capable of further elucidating the relationship between vector dispersal behavior and pathogen circulation, thereby assisting in the development of improved disease prevention and control measures.

## Introduction

The intensity of disease transmission to humans by mosquitoes, or vectorial capacity, depends, in part, on ecological parameters such as the feeding behavior of vectors on relevant vertebrate hosts, vector survivorship, dispersal patterns, and population density (1). Mark–release–recapture (MRR) is a standard approach to gather this epidemiologically significant information on mosquito behavior and ecology directly from field populations (2–4). Mosquito MRR studies have shaped our understanding of vector-borne disease transmission dynamics world-wide, comprising a body of literature of hundreds of studies on over 50 mosquito vectors of human pathogens (4). Among these studies, MRR targeting *Culex tarsalis* mosquitoes is among the most abundant (4).

Current marking techniques for mosquito dispersal studies include the use of topical fluorescent powders and paints (5), ingestible dyes (6), or larval habitat marking with rubidium (7) and, separately, stable isotopes.(8, 9) While fluorescent powders are widely used, they are limited by the difficulty of marking large numbers of mosquitoes, lack of powder retention, and impose negative effects on mosquito behavior and survivorship (5, 10, 11). Mosquitoes reared from larval habitats enriched in stable isotopes (12) or rubidium (7) can be detected via mass spectrometry or x-ray fluorescence spectrophotometry, respectively. However, these methods only provide a handful of distinguishable markers, and detection via mass spectrometry is expensive and training intensive. As an alternative to physically marking mosquitoes, recent investigations have also employed the use of single nucleotide polymorphisms (SNPs) and spatial genetic tools to estimate geographical dispersal of mosquitoes from the genetic relatedness of individuals (13, 14). While this approach is innovative and provides valuable insight into myriad mosquito population parameters, real-time field dispersal estimates are indirect, and specific expertise in generating and analyzing these types of data is required.

Here, DNA barcodes are short (∼100 to 200 bp) pieces of double-stranded DNA of known sequence representing the material's unique signature detected via sequencing (15). The feasibility of DNA serving as a tracking material has been tested in various applications (16). Surface-adsorbed DNA, in the form of silica-encapsulated DNA, has been studied as a tracking material for oils (17), trophic pathways (18), reservoir imaging (15), and aquifer characterization (19). Surface adsorption is suggested to afford nucleic acid resistance to nucleases (20). Advances in parallel synthesis and sequencing further promote DNA employment as MRR markers (21).

Recently, a hybrid fluorescent dye/DNA tag material was evaluated for mosquito marking (22). While the externally applied DNA tags of variable length remained robustly detectable up to 3 weeks, initial fluorescence-based recapture identification using ultraviolet (UV) light may degrade DNA tags. Here we develop a complementary self-marking strategy via ingestion.

**Fig. 1.**
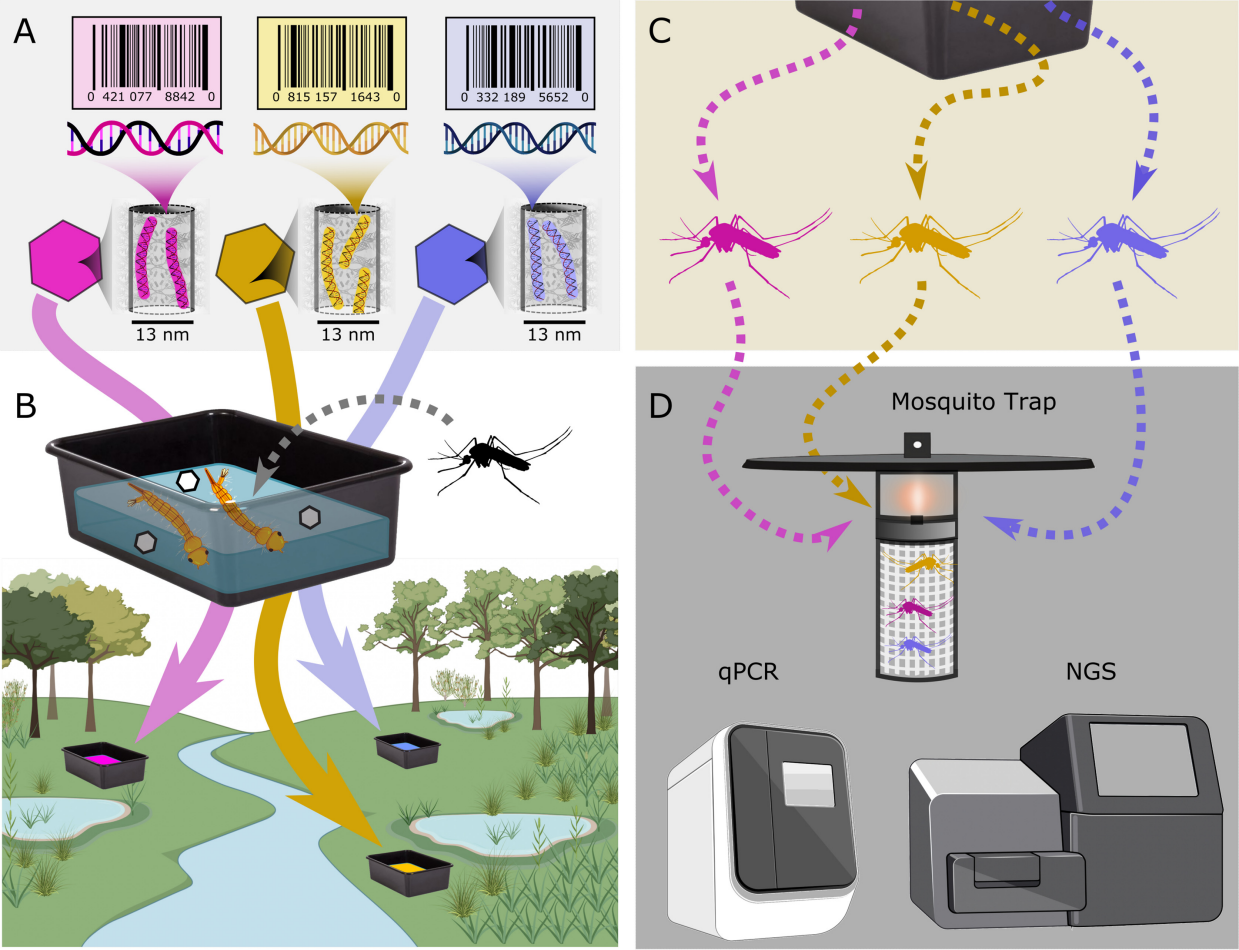
Mark–release–recapture strategy. (A) Synthetic DNA “barcode” sequences are designed, amplified, and loaded into the nanopores of engineered porous protein microcrystals. (B) Tubs are filled and placed at specific locations, dosed with specific DNA barcodes, and populated by mosquito larvae. (C) Trans-stadial persistence of the marker sequence in emerging adult mosquitoes allows for (D) detection of the recent origin of captured mosquitoes via qPCR or next-generation sequencing. Figure 1B was partially created on Biorender.com.

**Fig. 2.**
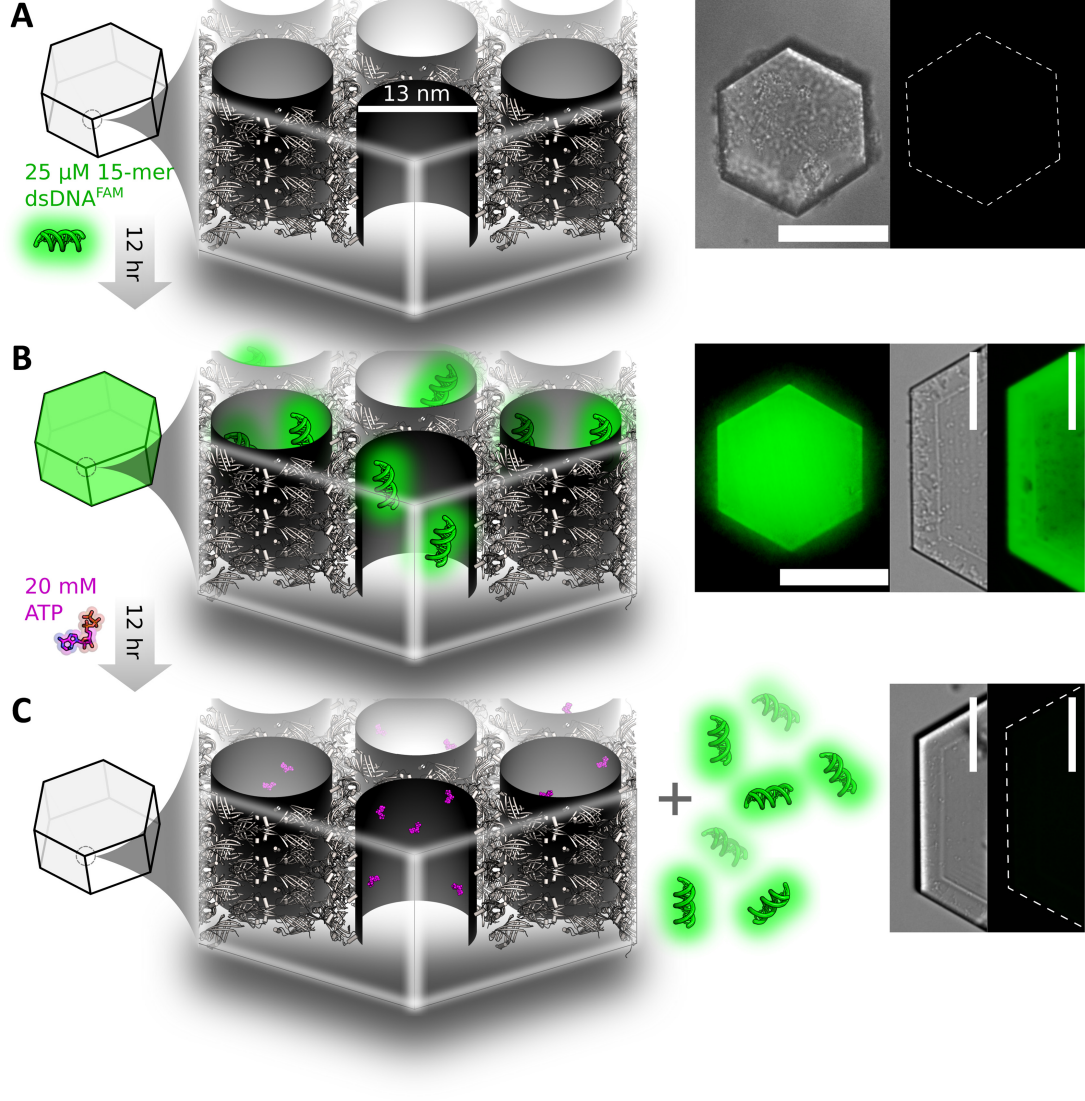
DNA loading and ATP-induced release. (A) Schematic of a CJ crystal pore, left, and a protein crystal imaged under bright-field and 488 nm excitation (right) revealing low background fluorescence of unloaded microcrystals. (B) Microcrystals following 12 h loading in a solution containing fluorophore-labeled (FAM) 15mer dsDNA. (C) Imaged microcrystals following 12 h incubation in a 20 mM ATP solution. Previously, fluorescent microcrystals exhibited almost no fluorescence due to nucleotide triphosphate-triggered release of microcrystal-adsorbed DNA. Scale bar denotes 100 μm.

We have identified a promising candidate material in DNA-loaded microcrystals that may overcome limitations inherent to conventional mosquito marking materials. Our central hypothesis is that porous protein microcrystals can carry and protect DNA barcodes following ingestion, and therefore be used to study movement patterns of field-collected mosquitoes (Fig. 1). Microcrystals composed of an isoprenoid binding protein “CJ,” from *Campylobacter jejuni*, feature an array of 13-nm-diameter pores suitable for DNA uptake (Fig. 2). Barcode DNA was designed using sequences not found in reference DNA sequence databases (Fig. S1) (23, 24). We then optimized the loading and recovery of DNA barcodes from cross-linked protein microcrystals and demonstrated persistent marking and barcode recovery from adult mosquitoes following oral ingestion as larvae. Furthermore, we have demonstrated that host microcrystals can confer some protection upon guest DNA from conditions that degrade free DNA. By overcoming previous limitations on marker diversity and encoding time and location data into a single barcode, this strategy allows data collection on vector movement patterns with a level of resolution that has been previously impossible.

## Results

### DNA loading and controlled release from host microcrystals

Following crosslinking, protein microcrystals exhibited negligible background fluorescence when imaged under 488 nm light (Fig. 2A). Following incubation in solution containing fluorescently labeled 15 bp double-stranded DNA (15mer), microcrystals appeared markedly fluorescent due to the strong retention of DNA adsorbed to the microcrystal interior despite two and three washes in buffer (Fig. 2B). The lack of observable DNA release during washing suggested that DNA was adsorbed with very high affinity. Consistent with strong noncovalent binding, it was possible to release guest DNA via incubation in solutions containing adenosine triphosphate (ATP) (Fig. 2C). Presumably, nucleotide triphosphate outcompeted microcrystal-adsorbed DNA for binding, resulting in the observed triggered release.

### Trans-stadial persistence of microcrystals from larval to adult stages

**Fig. 3.**
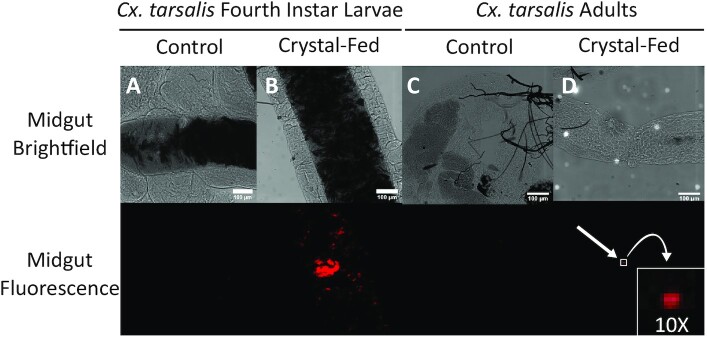
Detection of Texas-Red labeled microcrystals in the midgut of adult and larval mosquitoes. Composite panels show midgut images from both brightfield and fluorescence microscopy for *Cx. tarsalis* mosquitoes fed either liver powder alone (A and C), or liver powder supplemented with Texas-red-labeled protein microcrystals (B and D). Scale bar denotes 100 μm.

We first confirmed that larvae ingested microcrystals spiked into their aquatic environment and that ingested microcrystals could be localized within the larval mosquito digestive tract. After being fed non-DNA-loaded protein microcrystals conjugated with the fluorophore Texas Red throughout the second and third instar stages, *Cx. tarsalis* larvae were removed as fourth instar larvae for dissection. Confocal microscopy imaging of larval digestive tracts confirmed the presence of microcrystals (Fig. 3B) in 10/10 larval midguts examined, whereas microcrystals were absent in 10/10 control larvae fed only liver powder (Fig.3A). Fluorescent-tagged microcrystals were detected in all larvae fed a microcrystal-enhanced diet, although the density of microcrystals within digestive tracts varied by individual (Figs. S2 and S3). Preliminary observations indicated that microcrystals remained within the digestive tract, but work is still ongoing in this area. Microcrystal localization within the digestive tract followed no observable pattern, and microcrystals were roughly evenly dispersed throughout the foregut, midgut, and hindgut (Fig. S2). No microcrystals were visualized in the Malpighian tubules.

We next sought evidence that microcrystals ingested by mosquito larvae could persist trans-stadially to the adult stage. Remaining fourth instar larvae were allowed to pupate and eclose. Microcrystals were visualized within digestive tracts dissected from newly emerged adult female mosquitoes, demonstrating that microcrystals were retained in the alimentary tract of mosquitoes through development (Fig. 3C and D). Microcrystals ingested during larval development were not fully shed in the meconium; instead, a portion remained and could be visualized in the midgut post-eclosion. Microcrystals persisted trans-stadially in all 10 mosquitoes analyzed with this method. Importantly, barcode was detected in 82% (59 out of 72) of adult mosquitoes reared on barcoded microcrystals as larvae, and microcrystal ingestion did not affect survivorship of adult mosquitoes (Figs. S4 and S5, Table S1). We also confirmed delivery of microcrystals directly to adult mosquitoes via a sugar meal (Fig. S6).

### Barcode recovery and next-generation sequencing validation

**Fig. 4.**
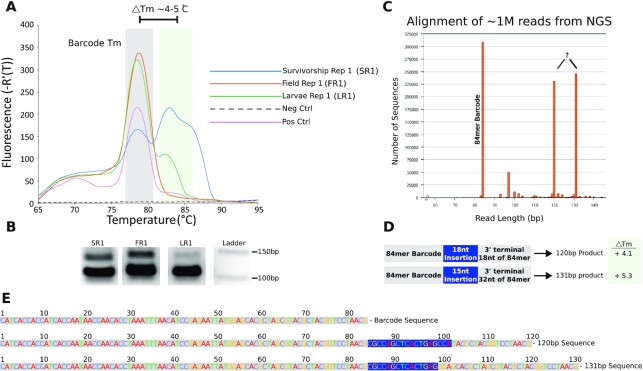
Barcode Detection. (A) qPCR melt curve displaying target 84-mer barcode peak in addition to a slightly higher peak (∼82 to 84°C) from samples obtained from survivorship, field, and larvae studies. For clarity, a single replicate from survivorship (SR1), field (FR1), and larvae (LR1) are shown. All replicates are shown in Fig. S11. (B) Gel electrophoresis following PCR amplification of samples shown in (A), have the expected target barcode band in addition to a weaker larger product band consistent with the qPCR melt curves. (C) Read length distribution for one million reads performed in Geneious Prime following next-generation sequencing of the samples shown in (B) reveal two prominent longer amplification products (120 and 131 bp), in addition to the target barcode sequence. (D) Inspection of the longer reads shown in (C) reveal a product that is still clearly barcode derived, albeit via an off-target concatenation event. The estimated *T*_m_ of the longer products was consistent with the higher melt peaks observed in (A). (E) Sequence composition of the intended barcode read and the longer products.

Mosquitoes that were exposed to microcrystals loaded with a synthetic barcode DNA sequence were subjected to homogenization and DNA extraction. Two primers were selected to amplify an 84-nt segment of synthetic barcode in qPCR experiments with three sample types. The first sample type included three lab colony mosquitoes (*Cx. tarsalis* Kern National Wildlife Refuge (KNWR) strain) that were fed barcode-laden microcrystals as larvae and from which barcode was detected in the emerged adult mosquitoes, designated as Survivorship Replicates 1 to 3 (SR1-3). The second sample type included three pools of wild-caught *Cx. tarsalis* and *Culiseta inornata* mosquitoes that colonized microcrystal-spiked tubs in the field and were later captured as adults in a CDC light trap, designated as Field Replicates 1 to 3 (FR1-3). The third sample type included three wild-caught *Cx. pipiens* mosquitoes that colonized microcrystal-spiked tubs placed in the field as larvae and were reared to adults in the laboratory, designated as Larvae Replicates 1 to 3 (LR1-3). The positive control was naked barcode DNA. The negative control was PCR master mix with no template added. The qPCR melt curves (Fig. 4A) had peaks corresponding to the target 84mer barcode peak (∼80.6°C) in addition to a slightly higher peak (∼82 to 84°C) observed among mosquitoes from the survivorship experiment as well as field-collected adult and larval specimens (Fig. S10).

To verify that the qPCR signal from the primary peak and shoulder peak were both resulting from an authentic barcode, size inspection of the PCR amplicons from samples shown in Fig. 4A was performed using gel electrophoresis (Fig. 4B). All the samples that were exposed to the synthetic barcode sequence had PCR amplified output products with the expected size for the 84-bp product. There was also a distinct and recurring band for an amplicon larger than expected (Fig. 4B). This larger product represented the observed higher melting temperature peaks (Fig. 4A).

We employed next-generation sequencing to further verify that the samples recovered from mosquitoes contained authentic barcode sequences, and that the side product that contributed to the qPCR signal was also derived from our synthetic barcode. The larger band in all lanes from the electrophoresis gel were extracted, prepared for sequencing by adding flanking Illumina adaptors using overhang PCR, and processed with next-generation sequencing (NovaSeq 6000).

In an analysis of one  million aligned reads (Fig. 4C), performed in Geneious Prime, the most common read corresponded to the expected 84-bp sequence, despite extracting the larger band size. The other two dominant read sequences corresponded to 120 and 131 bp sequences (Fig. 4D and E) that were almost entirely composed of the original synthetic barcode, with insertions/duplications suggesting an earlier off-target amplification event (e.g. mispriming) during the synthetic barcode amplification. The estimated *T*_m_ values (84.7°C and 85.9°C via Primer3 2.3.7) for these two longer products were consistent with the higher melt temperature region shown in Fig. 4A.

### Laboratory-reared mosquito barcode recovery and microcrystal protection

The persistence and recovery of synthetic DNA markers days after ingestion and metamorphosis was a remarkable result. We hypothesized that the host microcrystal would confer protection on guest DNA, thereby allowing guest DNA barcodes to survive in the mosquito midgut environment. In contrast, we expected naked DNA to be degraded (i.e. by nucleases). We further hypothesized that mosquito homogenate would be a similarly harsh environment for unprotected DNA. Microcrystal protection of loaded DNA in harsh solution conditions was directly tested by incubation of DNA-loaded microcrystals with filtered mosquito homogenate, where the guest DNA consisted of a 200-bp barcode sequence. As expected, DNA that was incubated in mosquito homogenate was only recovered if first loaded into microcrystals, as is evident by the presence of the PCR product band in lane 6 and the absence in lane 4 (Fig. S9C). DNA barcode was not detected in the negative control samples, nuclease-free water, and mosquito homogenate. The DNA barcode was recovered via PCR from the positive control samples, DNA in solution, and DNA-loaded microcrystals. Critically, these *in vitro* results were further supported *in vivo* through the elevated detection of barcode from adult mosquitoes fed DNA-loaded microcrystals relative to mosquitoes fed naked barcodes (Fig. S7). These results suggest that the primer sequences used do not cross-react with native mosquito homogenate (*Aedes aegypti*) and therefore do not lead to false-positive DNA detection at the estimated PCR product size of 200 bp. Furthermore, these results demonstrate plausible microcrystal protection of guest DNA from degradation under harsh solution conditions, allowing for downstream recovery and analysis via qPCR.

### Field trial collections and barcode detection

We made daily observations of larval development in both the 100 μL/week tub and the 1000 μL/week tub. As mosquito larvae presented in containers, pupae were removed, and reared to adulthood in the laboratory for crystal detection. This included 15 male *Cx. pipiens* sampled from the 1000 μL tub on August 6, and 9 *Cx. pipiens* from the 100 μL tub on August 13 (8 females and 1 male). From the four light traps, we established in each cardinal direction of the two bait stations (Fig. S10), we collected 34 *Cx. pipiens*, 172 *Cx. tarsalis*, and 1 *Culiseta inornata* for a total of 207 mosquitoes grouped into 55 pools. Results from this initial pilot study suggest barcode presence in approximately 76% (34 out of 45) of adult mosquito pools and 75% (18 out of 24) of larvae reared from the treated bins (Fig. S12). More extensive field evaluations are underway, and results will be reported elsewhere.

## Discussion

We have developed a new class of lightweight, durable marker particles composed solely of biomolecules (DNA and protein). These particles are suitable for persistently marking mosquitoes for ecological and biosurveillance applications. While these particles may also be suitable for topical application, the current study takes advantage of the size and intrinsic biocompatibility of the particles to assess the possibility of mosquito self-marking via particle ingestion. Microcrystal-adsorbed DNA was consistently trigger-released into the surrounding solution by ATP incubation (Fig. 2) and detected by common nucleic-acid amplification approaches (Fig. 4). Remarkably, DNA barcode-doped microcrystals ingested by mosquito larvae persisted through metamorphosis to the adult life stage (Fig. 3) without significantly affecting detection sensitivity (Fig. S8) or adult mosquito survivorship (Fig. S4). Moreover, barcode DNA adsorbed within protein microcrystals possessed an elevated resistance to degradation during *in vitro* mosquito homogenate incubation (Fig. S9C) and following ingestion by mosquito larvae (Fig. S6). We therefore propose that encasing DNA barcodes in the synthesized protein microcrystals provided some protection against barcode degradation by digestive enzymes or the basic pH (∼10 to 11) of the mosquito larval anterior midgut (25).

Our barcoding method is innovative in part due to the use of a designed synthetic DNA barcode strand that was easily recovered from individual mosquitoes or from pools. Specifically, the barcode sequence used in this study exploited nullomer sequences (24) not found in publicly available databases to label mosquitoes (Fig. S1). The second major innovation is the protection of potentially vulnerable DNA in the interior of crosslinked porous protein microcrystals. To this end, we take advantage of our fortuitous empirical discovery that DNA strongly adsorbs to the interior of our porous protein microcrystals (26). The resulting doped microcrystals could be easily integrated into ongoing mosquito surveillance activities, for both research and operational purposes. Larval habitats, particularly artificial containers, can be uniquely marked by the addition of DNA-doped microcrystals (Fig. 1 and Fig. S10). Barcodes can then be amplified from mosquito pools already undergoing arbovirus testing, thereby linking information on spatial and temporal movement patterns to virus transmission cycles. Extrinsic barcode recovery will directly indicate visited locations for captured mosquitoes, including those that are disease vectors.

While qPCR provides a highly sensitive method to quantify DNA based on the generation of a fluorescence signal, it does have certain limitations when working with environmental field samples. Due to a lack of sequence specificity in SYBR Green-based assays, the presence of non-template DNA and/or template fragments may cause heterogenous melt curves, reducing confidence in positive detection events. While TaqMan qPCR offers sequence specificity, such assays require costly probes and are limited in channel-based multiplexing capability, allowing less than 10 samples to be run in parallel (27), excluding recent fluorescence modulation-based approaches, which consume more probe per assay, thus increasing sample processing cost (28). Moreover, multiplexing qPCR requires optimization of primer sequences, requiring additional time in assay design. In contrast, high-throughput next-generation sequencing offers direct read confirmation of target template with nucleotide resolution, eliminating ambiguity in barcode detection from observed qPCR melt curves, while allowing for multiplexing at a scale (hundreds) presently inaccessible by current probe-based qPCR methods.

An additional limitation of the present study is the barcode design. The barcode was obtained as a singular, full-length sequence (125 bp), and PCR was used to synthesize barcode stock for subsequent experiments. Purchasing each barcode sequence is not a particularly scalable approach, considering the potential demand for hundreds of barcodes to represent different field sample sites and times. A more modular and scalable barcode DNA production method is therefore advisable for future studies that differentiate multiple sample sites, and therefore have the potential to provide insight on the degree of connectivity between isolated locations and how that connectivity evolves temporally by taking weekly samples. In particular, the use of barcodes at multiple field sites may allow investigators to understand the degree of habitat sharing and gene flow occurring between mosquitoes originating from different locations. Additional resolution to such data may be introduced by dosing field sites weekly with new, unique barcodes while simultaneously trapping for adult mosquitoes.

In conclusion, DNA-barcoded microcrystals represent an advanced functional biomaterial, and an innovative technology platform for studying mosquito dispersal, and subsequently, arbovirus circulation by vectors in the field. We have demonstrated proof-of-concept of this technology in the laboratory and in a small pilot field study. Laboratory investigation has shown that (1) *Culex* mosquitoes can be orally marked as larvae by spiking the larval habitat with DNA-loaded microcrystals, thereby providing a self-marking strategy for mosquitoes in the field, (2) microcrystals persist trans-stadially through mosquito development, (3) ingestion of microcrystals by mosquito larvae does not affect adult mosquito survival or development, and (4) encoded information can be recovered from mosquitoes by qPCR and NGS, with detection sensitivity unaffected by mosquito pool size. This latter result is directly translational to vector surveillance activities in which mosquito pools would also be tested for arbovirus nucleic acid.

One consideration left to be addressed prior to extensive field deployments is the larval feeding behavior of the target mosquito species (29). This approach may be more conducive to mosquito species that dive and filter particles suspended in the water column (i.e. *Culex*, spp. the focus of these initial studies), as opposed to those species that feed at the surface or are predaceous. Additionally, unit cell calculations from Kowlaski et al. show that microcrystals are approximately 80% solvent due to high porosity, allowing them to readily equilibrate with the surrounding solution and sink to the bottom, resulting in a dynamic availability to mosquitoes (30). While this approach may not function as a direct substitute for research questions requiring knowledge of the marked population size, new doors are opened for gathering more applied information on mosquito populations that may be operationally significant. Collectively, these findings represent the critical first steps to implementation of this technique into mainstream vector research and surveillance activities. Lastly, it is important to consider the possibility of marked mosquitoes transferring DNA barcode to larval and adult mosquitoes that have not previously visited a bait station. Such lateral transfer, as previously described (31), would frustrate data analysis leading to inaccurate conclusions in studies examining population size or dispersal, for example. Future work aims to address this possibility.

## Materials and methods

### Porous microcrystal production and fluorophore labeling

Microcrystal-forming protein derived from *Campylobacter jejuni* was expressed, purified, and crystallized as described previously (PDB entry 5w17)(30, 32). Microcrystals were crosslinked using 1-ethyl-3-(3-dimethylaminopropyl) carbodiimide hydrochloride (EDC) or glyoxal during trace-labeling with Texas Red dye (ThermoFisher) as described previously (33). See Supplementary Material (Porous Crystal Production, Crystal Fluorophore Labeling) for additional details regarding microcrystal growth, crosslinking, and labeling.

### DNA loading and ATP-induced DNA release

Cross-linked microcrystals were placed in 100 µL TE buffer (10 mM Tris, 1 mM EDTA, and pH 7.4) for 1 hour to equilibrate microcrystal pores for DNA loading. Microcrystals were imaged under 488 nm excitation with a fluorescence confocal microscope (Nikon Eclipse Ti-E) equipped with an Andor iXon DU-897 EMCCD camera to establish baseline fluorescence of non-DNA-loaded microcrystals. Microcrystals were then immersed in 10 µL of 25 µM 15 base pair double-stranded DNA,15mer (5′—CCGCACGCACGAGGC–3′) labeled with 6-FAM (fluorescein) at the 5′ terminus (IDT), and sealed in a glass well plate for approximately 12 hours. Following DNA loading, microcrystals were washed with TE buffer to remove unbound 15mer. Microcrystal retention of DNA was confirmed by fluorescence imaging. To trigger release of microcrystal bound 15mer, microcrystals were immersed in 20 mM ATP in TE buffer, and sealed in a glass well plate for approximately 12 hours. Microcrystals were then imaged by fluorescence confocal microscopy, confirming ATP-induced DNA release by a reduction in microcrystal fluorescence following ATP incubation.

### Trans-stadial persistence of microcrystals from larval to adult stages

Colony-reared, KNWR strain *Cx. tarsalis* were hatched in 9oz clear, plastic cups under standard insectary conditions (34). Briefly, eggs were hatched in water to produce roughly 100 first-instar larvae in each cup. Control larvae were fed 250 µL of 10% liver powder solution each day, while microcrystal-exposed larvae were fed 225 µL liver powder solution thoroughly mixed with 25 µL non-DNA-loaded microcrystals conjugated with the fluorophore Texas Red and suspended in water. At the fourth instar stage, control and microcrystal-fed larvae (*n* = 10 for each) were removed for dissection. A portion of both larvae groups were allowed to pupate and emerge as adults. Immediately after emergence, female *Cx. tarsalis* (*n* = 10) were removed for cold-induced knock down and dissection. Whole digestive tracts were dissected from larvae and adults in PBS and then mounted onto slides with ProLong Gold Antifade Mountant with DAPI. Slides were stored in the dark at −20°C for at least 24 hours prior to confocal microscopy. Larvae and adult dissections were imaged with a Nikon Eclipse Ti-E microscope equipped with an Andor iXon DU-897 EMCCD camera at 10X magnification under brightfield and 561 nm excitation (10% laser power).

### In vivo qPCR barcode recovery

Following extraction, qPCR was performed using 6 µL of each sample as the template for 20 µL reactions with the following primers: fwd 5′—CATCACCACCATCACCAA–3′, rev 5′—CGTTAGGACCGTAGCGTA–3′. Primers used were designed to amplify an 84 bp subsequence of the 125 bp synthetic barcode (84 mer) initially loaded into microcrystals. Primers were designed using Primer3 (35, 36) to enhance template amplification while reducing marked primer-dimer formation observed with an initial primer set (Primer1_114F, Primer1_114R) adopted from Goswami et al (24). Standards used in qPCR consisted of serial dilutions of the PCR amplified 84mer. The quantitative amplification was performed using the manufacturer's guidelines (Agilent qPCR Brilliant II SYBR Master Mix). Reaction conditions were: 1 cycle of 95°C for 3:00 min and 45 cycles of 95°C for 5 s and 60°C for 10 s. Melt curve was obtained by 1 cycle of 95°C for 30 s, 65°C for 30 s and 95°C for 30 s. To obtain a quantitative cutoff for the qPCR data, we fit each curve as a sum of Gaussian functions using LMFIT (37) and a custom Python script to constrain the center position and width of the component Gaussian functions. On the whole, the resulting functions fit the data remarkably well (Figs. S5 and S12). To provide a conservative cutoff we only classified datasets as “positive” for barcode if the peak height of the ∼78°C Gaussian exceeded the height of the neighboring Gaussian (∼74°C) by a factor of 1.5 and possessed a raw height value greater than 10 (background). Raw data and Python code available on Zenodo (DOI: 10.5281/zenodo.6834837).

### Next-generation sequencing

Barcode positive samples from survivorship and field studies detected via qPCR were prepared for sequencing by using 1µL of each field sample as the template for overhang PCR to append Illumina sequencing primer binding sequences using the following primers: fwd 5′—ACACTCTTTCCCTACACGACGCTC TTCCGATCTCATCACCACCATCACCAA–3′, rev 5′—GTGACTGGAG TTCAGACGTGTGCTC TTCCGATCTCGTTAGGACCGTAGCGTA–3′. Thermocycling conditions for overhang PCR were: 1 cycle at 98°C for 2:00, 2 cycles at 98°C for 20 s, 58°C for 30 s, 72°C for 30 s, and 1 cycle at 72°C for 1:00. An additional overhang PCR was performed to append Illumina flow cell hybridization sequences using the following primers: fwd 5′—AATGATACGGCGACCACC GAGATCTACACTCTTTCCCTACACGACGCTCTTCCGATCT–3′, rev 5′—CAAGCAGAAGAC GGCATACGAGATNNNNNNNNNNATATTCA CGTGACTGGAGTTCAGACGTGTGCTCTTCCGATCT–3′. Thermocycling conditions for additional overhang PCR were: 1 cycle at 98°C for 2:00, 2 cycles at 98°C for 15 s, 63°C for 30 s, 72°C for 30 s, and 1 cycle at 72°C for 1:00. Lastly, PCR was performed on the full-length template using the following primers: fwd 5′—AATGATA CGGCGACCACCGAGATCT–3′, rev 5′—CAAGCAGAAGACGGCATACGAGAT–3′. Thermocycling conditions PCR were: 1 cycle at 98°C for 2:00, 30 cycles at 98°C for 15 s, 58°C for 30 s, 72°C for 30 s, and 1 cycle at 72°C for 1:00. Following each amplification, PCR cleanup was performed using KAPA Pure Beads (Roche). Size selection for the 218 bp barcode library was performed using Monarch DNA Gel Extraction Kit (New England Biolabs). The library was quantified using Qubit 1X dsDNA HS Assay Kit (ThermoFisher) and diluted to 20 nM for sequencing sample prep. Paired end 2 × 150 cycle sequencing was run on an Illumina NovaSeq 6000 (Genomics and Microarray Core, University of Colorado Anschutz Medical Campus). The ea-utils package was used for initial sample processing, including adapter trimming and read joining. FastQC was used to check overall quality of joined reads and to determine total read count of detected barcode (Babraham Bioinformatics). Alignment was performed using Geneious Prime 2021.0.1.

### Mosquito processing for barcode detection

Mosquito pools were homogenized by addition of 1 mL mosquito diluent (Dulbecco's Modified Eagle Medium (DMEM) with 20% fetal bovine serum (FBS), 50 µg/mL penicillin/streptomycin, 50 µg/mL gentamicin, and 2.5 µL/mL fungizone), two glass Coliroller beads (Novagen), and 75 µL of 100 mM ATP, and homogenized for 3 min at 24 Hz using a Retsch Mixer Mill (Retsch). Nucleic acid extractions were performed with MaxMAX Cell-Free DNA extraction kit, using a modified protocol (see Supporting Information—Barcode Recovery from Homogenized Mosquitoes) in a 96-well plate format on a Kingfisher Flex automated extraction platform (Thermo Fisher). qPCR was performed using PowerUp SYBR Green Master Mix (Thermo Fisher), with the primers, cycling conditions, and standard quantification described above for in vivo barcode recovery.

### Pilot field trial

Two black 18 Qt Sterlite wash basins were set outdoors on the CSU Foothills Campus and filled with stagnant organic-rich water taken from the field site to mimic a natural mosquito larval habitat. One tub received 1000 µL of microcrystal stock (weekly) and the other received 100 µL of microcrystal stock weekly for four weeks during July to August 2020. Containers were topped up daily to offset evaporation. When mosquito larvae naturally colonizing these tubs began to pupate, four Centers for Disease Control light traps (John W. Hock) were operated nightly to capture emergent mosquitoes. Additionally, representative fourth instar mosquito larvae or pupae were removed from the tubs and reared to adults in the insectary for the purposes of confirming the presence of barcode DNA. Pooled and individual mosquitoes were processed for barcode detection as described above.

### Barcode protection by microcrystals

To demonstrate the extent to which microcrystals protect PCR-recoverable DNA in a complex matrix, a 50 µL solution containing crosslinked microcrystals in TE buffer was incubated with 50 µL of 10 ng/µL ∼200 bp double-stranded DNA solution (200mer) for 12 hours. Following DNA loading, microcrystals were washed in fresh TE buffer using Amicon Ultra-15 centrifugal filter units (Millipore Sigma) at 4700 RPM for 5 min (3x). DNA-loaded microcrystals were incubated with an equal volume of filtered mosquito homogenate for 12 hours, followed by addition of 20 mM ATP for another 12-hour incubation. The 200mer/microcrystal/homogenate solution was then used as the template DNA for PCR (forward primer: 5′-AATGATACGGCGACCACCGAGATCT-3′, reverse primer: 5′-CAAGCAGAAG ACGGCATACGAGAT-3′) using Q5 High-Fidelity DNA Polymerase (NEB) with the following cycling conditions: 1 cycle at 98°C for 45 s, 30 cycles at 98°C for 15 s, 55°C for 30 s and 72°C for 30 s, and 1 cycle at 72°C for 60 s. The negative control samples consisted of nuclease-free water and, separately, mosquito homogenate. Positive control samples included 200mer in solution and 200mer retrieved from loaded microcrystals, both in the absence of mosquito homogenate. DNA recovery was assessed by 5% agarose gel electrophoresis.

## Supplementary Material

pgac190_Supplemental_Files

## Data Availability

Raw data and Python code available on Zenodo (DOI: 10.5281/zenodo.6834837).
