# Supplementary material for: Mosquito tagging using DNA-barcoded nanoporous protein microcrystals
Source: PNAS Nexus. 2022 Sep 12;1(4):pgac190. doi: 10.1093/pnasnexus/pgac190 (PMC9802479; doi:10.1093/pnasnexus/pgac190)
Supplement: pgac190_Supplemental_Files [file pgac190_supplemental_files.zip › PNASNEXUS-PNASNEXUS-2022-00358-T-s01.pdf]

Supplemental Information for
Mosquito Tagging Using DNA-Barcoded Nanoporous Protein Microcrystals

Authors

Julius D. Stuart[a,1], Daniel A. Hartman[b,c,1], Lyndsey I. Gray[b], Alec A. Jones[d], Natalie R. Wickenkamp[b], Christine Hirt[b,e], Aya Safira[d,f], April R. Regas[g], Therese M. Kondash[h,i], Margaret L. Yates[j], Sergei Driga[k], Christopher D. Snow[a,d,j,k], Rebekah C. Kading[b]

Author Affiliations

[a]Department of Chemistry, Colorado State University, Fort Collins, CO 80523; [b]Department of Microbiology, Immunology, and Pathology, Colorado State University, Fort Collins, CO 80523; [c]Department of Entomology, Cornell University, Ithaca NY 14853 (current);[d]School of Biomedical Engineering, Colorado State University, Fort Collins, CO 80523;[e]Invitae, Longmont, CO 80503 (current);[f]Just-Evotec Biologics, Seattle WA 98109 (current);[g]College of Veterinary Medicine and Biological Sciences, Colorado State University, Fort Collins, CO 80523;[h]Department of Environmental Health and Radiological Sciences, Colorado State University, Fort Collins, CO 80523; [i]H3 Environmental, Albuquerque, NM 87109 (current);[j]Department of Biochemistry and Molecular Biology, Colorado State University, Fort Collins, CO 80523; [k]Department of Chemical and Biological Engineering, Colorado State University, Fort Collins, CO 80523

Corresponding Author

Rebekah C. Kading
(970) 491-7833
Rebekah.Kading@colostate.edu
176 CVID
Colorado State University
Fort Collins, CO 80523

[1]J.D.S. and D.A.H. contributed equally to this work.

This PDF file includes:

DNA and protein sequence content for crystal loading and recovery
Figures S1 – S8
Table S1

DNA Sequence (5’ – 3’) for Microcrystal Protein Monomer
TTAAGAAGGAGATATACATATGAAAAAAGTTCTGCTGAGCAGCCTGGTTGCAGTTAGCCTGCTGAGTACCGGTCT
GTTTGCAAAAGAATATACCCTGGATAAAGCCCATACCGATGTTGGCTTTAAAATCAAACATCTGCAGATTAGCAAT
GTGAAAGGCAACTTTAAAGATTATAGCGCAGTGATCGATTTTGATCCGGCAAGTGCAGAATTCAAAAAACTGGAT
GTGACCATTAAAATCGCCAGCGTGAATACCGAAAATCAGACCCGTGATAATCATCTGCAGCAGGATGACTTCTTCA
AAGCCAAAAAATACCCGGATATGACCTTTACCATGAAAAAATACGAGAAAATCGATAACGAAAAAGGCAAAATGA
CCGGCACCCTGACCATTGCCGGTGTTAGCAAAGATATTGTTCTGGATGCAGAAATTGGTGGTGTTGCCAAAGGTA
AAGATGGCAAAGAAAAAATTGGCTTTAGCCTGAACGGCAAAATCAAACGTAGCGATTTCAAATTTGCAACCAGCA
CCAGCACCATTACCCTGAGTGATGACATTAATCTGAACATTGAAGTGAAAGCCAACGAGAAAGAAGGTGGTAGTC
ATCACCACCACCATCACTAATAACTCGAGCACCACCACCACCACCACTGAGATCCGGCTG

Protein Sequence for Microcrystal Protein Monomer
MKEYTLDKAHTDVGFKIKHLQISNVKGNFKDYSAVIDFDPASAEFKKLDVTIKIASVNTENQTRDNHLQQDDFFKAKKYP
DMTFTMKKYEKIDNEKGKMTGTLTIAGVSKDIVLDAEIGGVAKGKDGKEKIGFSLNGKIKRSDFKFATSTSTITLSDDINL
NIEVEANEKEGGSHHHHHH

200mer sequences (5’ – 3’)
Nuclease-free water sample:
AATGATACGGCGACCACCGAGATCTACACTCTTTCCCTACACGACGCTCTTCCGATCTTACTAGGCGACTCGACGGT
CTTACGCGTTACGTCCGACTATAGAGCTTAGATTAGCGACGTTAAGATCGGAAGAGCACACGTCTGAACTCCAGTC
ACACAGGCGCNNNNNNNNNNATCTCGTATGCCGTCTTCTGCTTG
Mosquito homogenate sample:
AATGATACGGCGACCACCGAGATCTACACTCTTTCCCTACACGACGCTCTTCCGATCTATACTAGACCGCTCGATCC
GACCTAGCGTACCTAGTACGTTACGACGACTAAGCATACCGCTAAGATCGGAAGAGCACACGTCTGAACTCCAGT
CACCATAGAGTNNNNNNNNNNATCTCGTATGCCGTCTTCTGCTTG
Loaded microcrystals in nuclease-free water sample:
AATGATACGGCGACCACCGAGATCTACACTCTTTCCCTACACGACGCTCTTCCGATCTCTCTCGTCCGACGGTCTTA
CGCGTTACGCCAAGTCTGCTAGCGTACGCTACGGTCTTGGACTCAGATCGGAAGAGCACACGTCTGAACTCCAGTC
ACTGCGAGACNNNNNNNNNNATCTCGTATGCCGTCTTCTGCTTG
Loaded microcrystals in mosquito homogenate sample:
AATGATACGGCGACCACCGAGATCTACACTCTTTCCCTACACGACGCTCTTCCGATCTAGCAGAATTCGACGGTCTT
ACGCGTTACGATGAGGCCGCTAGCGTACGCTACGGTCACTAAGATAGATCGGAAGAGCACACGTCTGAACTCCAG
TCACTCTCTACTNNNNNNNNNNATCTCGTATGCCGTCTTCTGCTTG

200mer forward primer DNA sequence (5’ – 3’)
AATGATACGGCGACCACCGAGATCT
200mer reverse primer DNA sequence (5’ – 3’)
CAAGCAGAAGACGGCATACGAGAT

125mer DNA Sequence (5’ – 3’)
TAGGCGACTCGACGGTCTTACGCGTTACGTATGATATGCATCACCACCATCACCAATAACCAACACCTAAATTTAAC
ATCCGAGAATTATGGAGCACGCTAGCGTACGCTACGGTCCTAACGCGC

125mer forward primer DNA sequence (5’ – 3’)
TAGGCGACTCGACGGTCTTACGCGTTACGT
125mer reverse primer DNA sequence (5’ – 3’)
GCGCGTTAGGACCGTAGCGTACGCTAGCGT

125mer revised forward primer DNA sequence (5' – 3')
CATCACCACCATCACCAA

15mer TAMRA DNA Sequence (5' – 3')
TAMRA - CGGAGCACGCACGCC

15mer Fluorescein DNA Sequence (5' – 3')
FAM - CCGCACGCACGAGGC

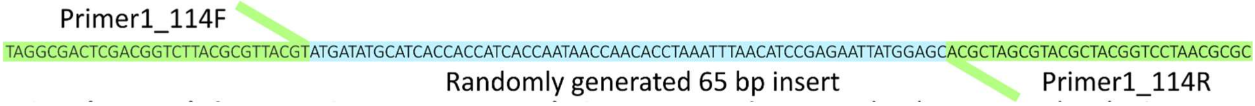

Figure S1. DNA Barcode Sequence Design

A randomly generated 65 bp insert using a publicly available online Sequence Manipulation Suite(1) is flanked by nullomer barcode primers provided in the Supplemental Information for Goswami et al. and follow the same naming convention(2).

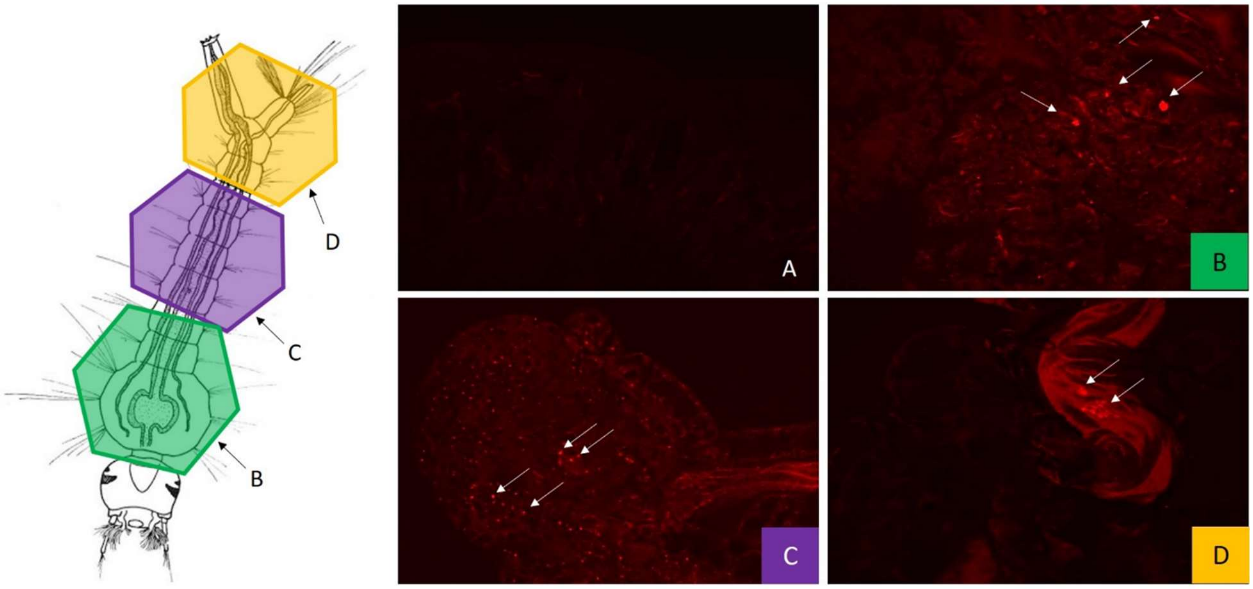

Figure S2. Detection of Texas Red labeled crystals in multiple regions (panels **B – D**) of the larval midgut of *Culex tarsalis* mosquitoes corresponding to labeled regions in diagram (left). No fluorescence was detected from non-crystal fed larvae (panel **A**). Scale bar not shown.

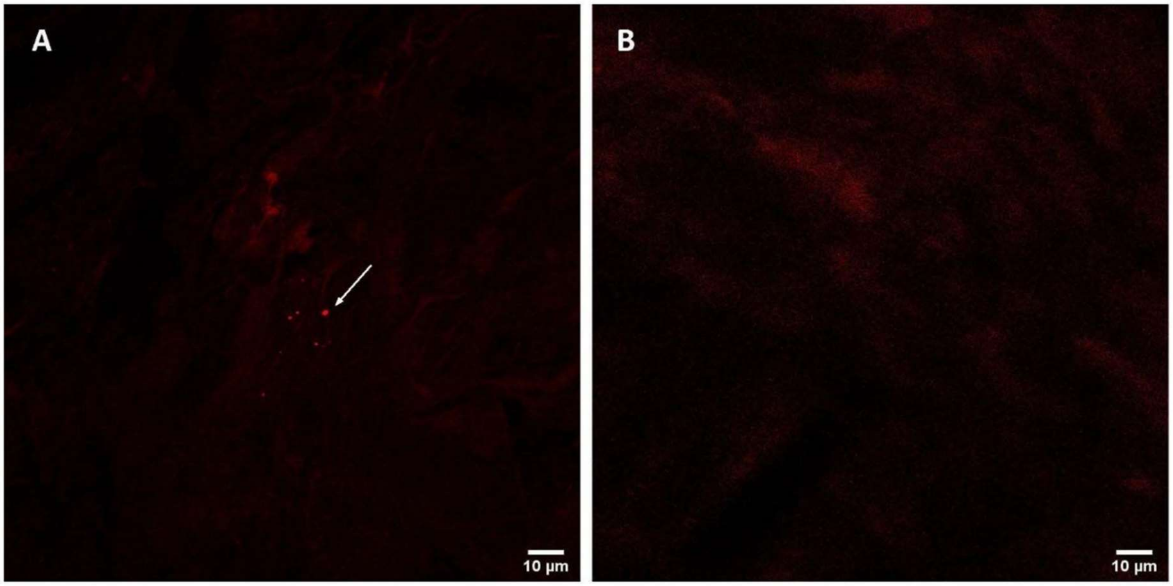

Figure S3. A) Microcrystals loaded with Texas Red visualized inside the alimentary tract of larval *Culex tarsalis* mosquitoes. **B)** Negative control: *Culex tarsalis* larval alimentary tract after consuming liver powder alone. Images taken at 40X magnification. Scale bar denotes 10 µm.

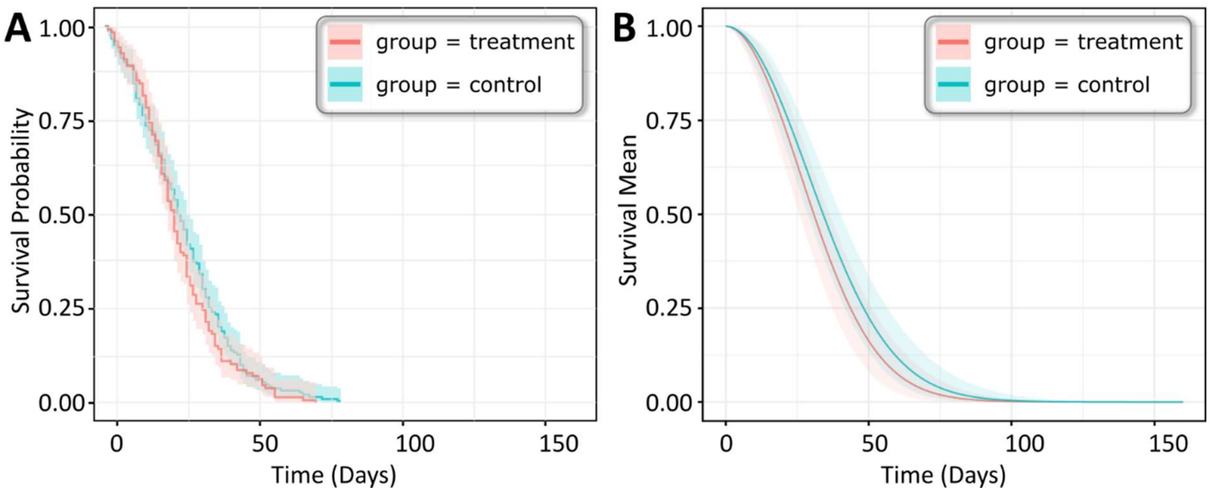

Figure S4. A) Kaplan-Meier curves for two combined replicates of adult mosquito survivorship. Lines indicate survival probabilities for treatment (larvae fed microcrystals mixed with liver powder) and control (larvae fed liver powder alone) mosquito groups. Shaded areas indicate 95% confidence intervals around survival probabilities. **B)** Posterior predictions for adult mosquito survival probabilities from the Weibull survival model are shown as lines for treatment and control groups. Plotting the posterior predictions show overlap between 95% credible intervals over the entire time course.

Table S1. Temporal Analysis of Survivorship Barcode Detection. Overall, barcode detection exhibits a decreasing trend with age. However, the majority of mosquitoes remained barcode-positive out to 40 days post-emergence, suggesting an elevated likelihood of barcode persistence for the mosquito lifetime and subsequent recovery.

Adult Mosquito Age	% barcode positive	n
1-10	100	14
11-20	73	26
21-30	81	21
31-40	83	6

Microcrystals do not affect adult mosquito survival or development

Larval *Culex tarsalis* mosquitoes reared on either liver powder alone (control) or liver powder with the addition of DNA barcoded microcrystals (treatment) were reared to adulthood and tracked individually for length of survivorship in days. Of the two replicates completed, the adult survivorship of mosquitoes was not significantly different between control and treatment groups. Crystal-fed mosquitoes across all replicates lived an average of 24 ± 13 days as adults (n=125), with the longest-lived mosquito reared on microcrystals surviving 67 days. By comparison, control mosquitoes survived an average of 26 ± 19 days as adults (n=177). One mosquito in the control group survived 196 days, a clear outlier; the next longest-lived control mosquito survived 69 days. qPCR of individual mosquitoes from the treatment group suggests an approximate 82 % barcode recovery rate, based on analysis (fig. S5) of melt curve data (n = 72). Preliminarily, a significant effect on larval survivorship was observed ($p = 2.747e-05$, Fisher's Exact Test) between larvae reared on microcrystals plus liver powder as opposed to those fed liver powder alone. However, this warrants further evaluation as only two replicates were available for this analysis and the effect of larval density was not considered but may have also affected larval survivorship.

Figure S5. Melt curves and scoring results for qPCR of mosquito extractions from survivorship experiments (1/2). Representative melt curves for individual mosquitoes from survivorship experiments. T1 and T2 denote replicate group 2 and 3, respectively, followed by individual replicate number. Melt curves containing a peak at ~78.5 °C with a height at least 50% greater than the neighboring peak at ~ 74 °C using the python package LMFIT(3) were scored as positive. Left plots display raw qPCR melt curve data overlaid by the obtained fit along with the corresponding residuals. Right plots display raw qPCR data overlaid with the set of obtained gaussian peaks from computational analysis. The purple peak represents the detected DNA barcode. Analysis results of all samples are found on Zenodo(DOI: 10.5281/zenodo.6834837).

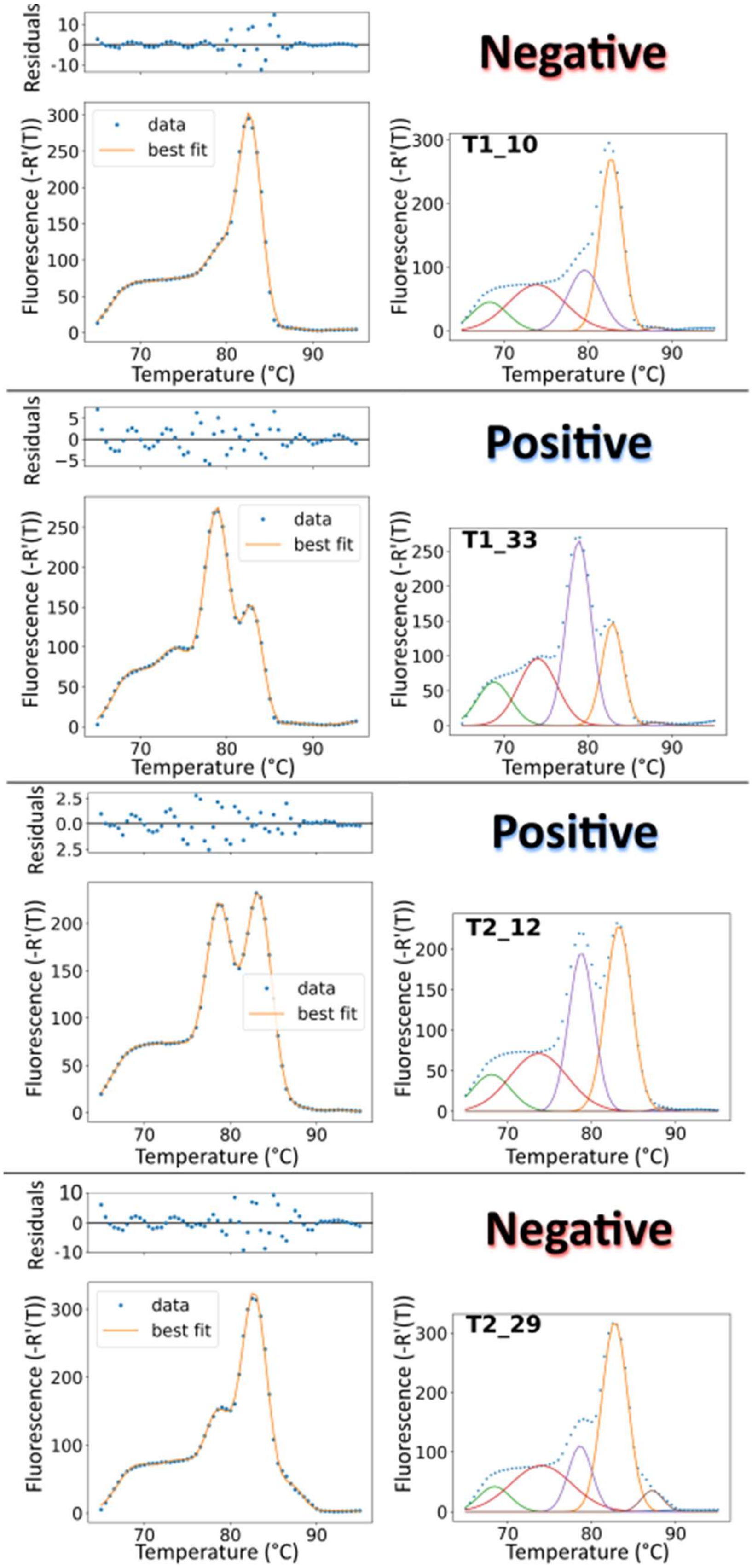

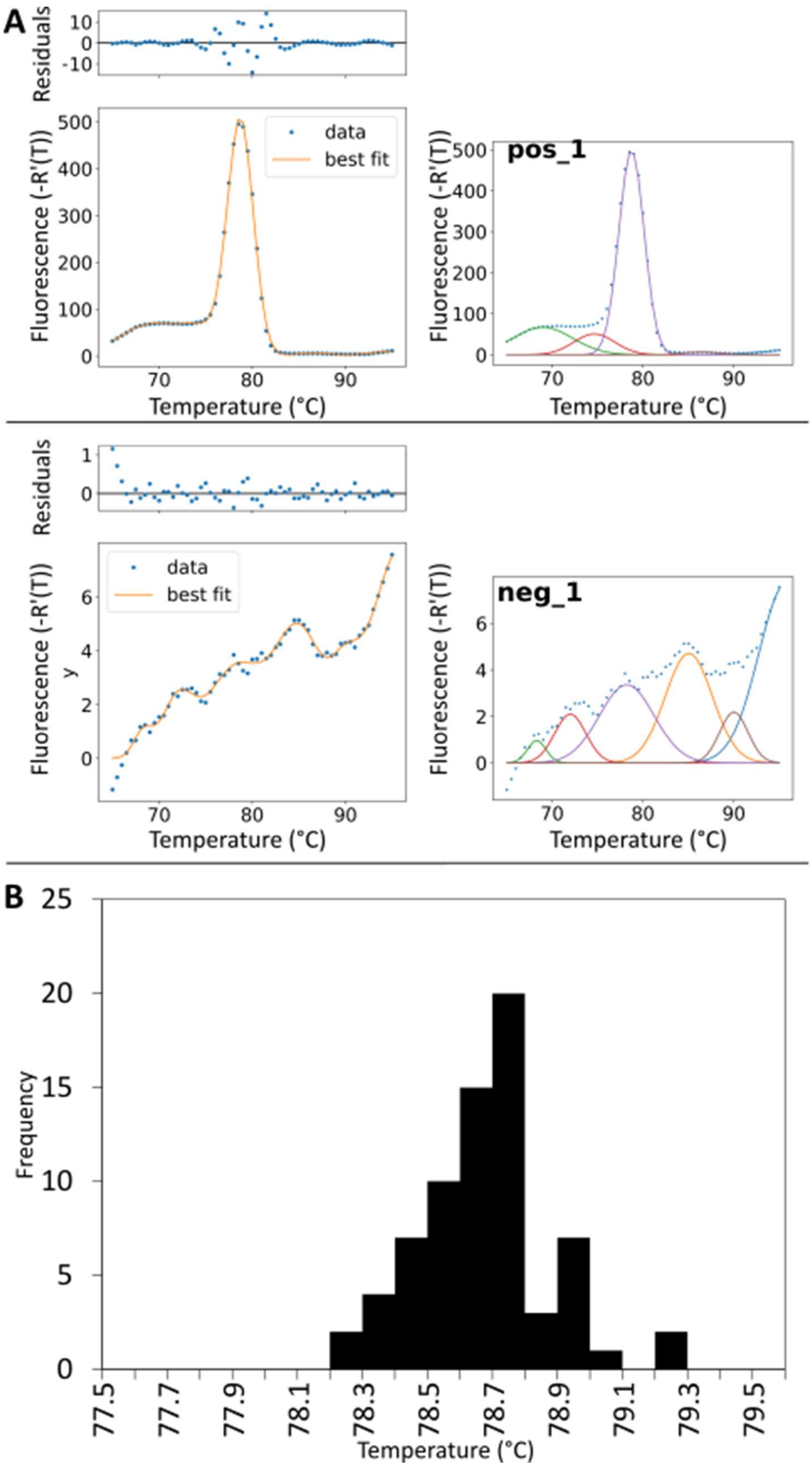

Figure S5. Melt curves and scoring results for qPCR of mosquito extractions from survivorship experiments (2/2). (A) Melt curves for a positive control (top) and negative control (bottom) overlaid with LMFIT(3) results. **(B)** Histogram of fitted barcode peak centers for all 72 samples revealing a narrow distribution for identified barcode peaks.

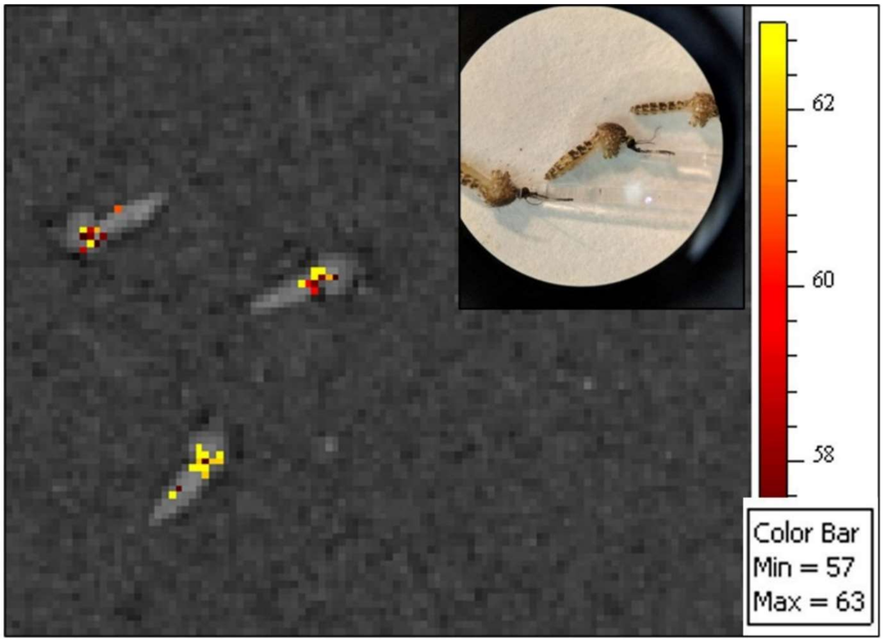

Figure S6. Ingestion of fluorescein-labeled crystals by adult *Culex tarsalis* mosquitoes. Mosquitoes were immobilized by removal of legs and wings. The proboscis of each mosquito was inserted into a capillary tube containing microcrystals in sugar solution. After approximately 30 minutes of feeding, mosquitoes were live-imaged using an In Vivo Imaging System (IVIS). A fluorescent signal is present inside the mosquito body indicating that crystals were ingested.

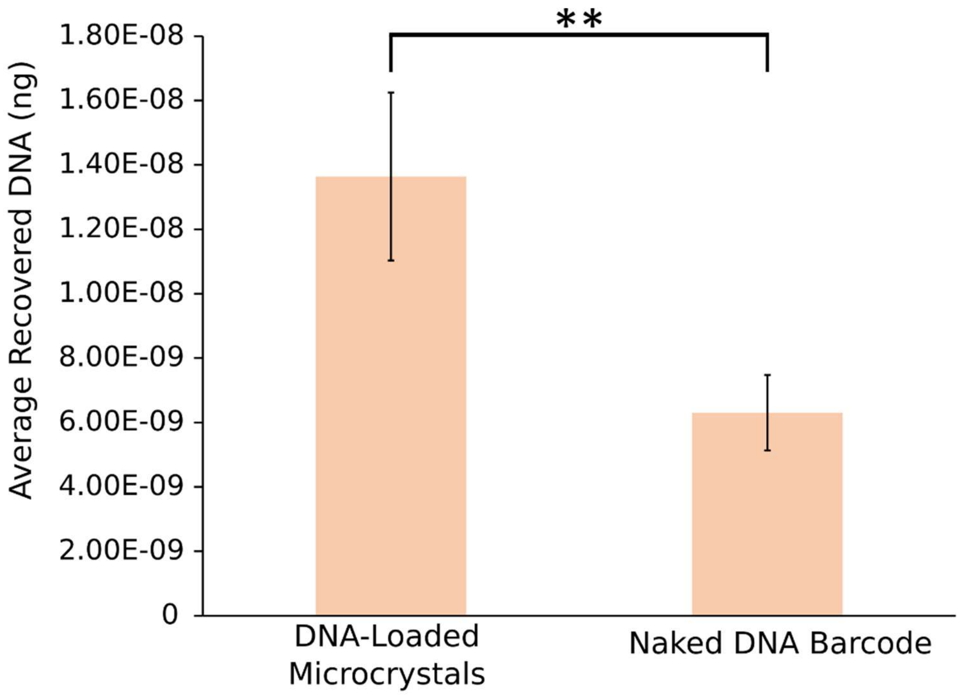

Figure S7. Environmental Persistence. Bar chart showing elevated DNA barcode recovery from mosquitoes fed DNA-loaded microcrystals mixed with liver powder (n = 36) relative to mosquitoes fed naked DNA barcode in liver powder solution (n = 45). Mosquito larvae were fed 6.5 ng of either naked barcode or barcoded microcrystals daily during the 2nd, 3rd, and 4th instar life stages. Adult mosquitoes were harvested upon emergence for DNA extraction and barcode detection as described in the Materials and Methods. Error bars represent one standard deviation ($p = 0.0075$).

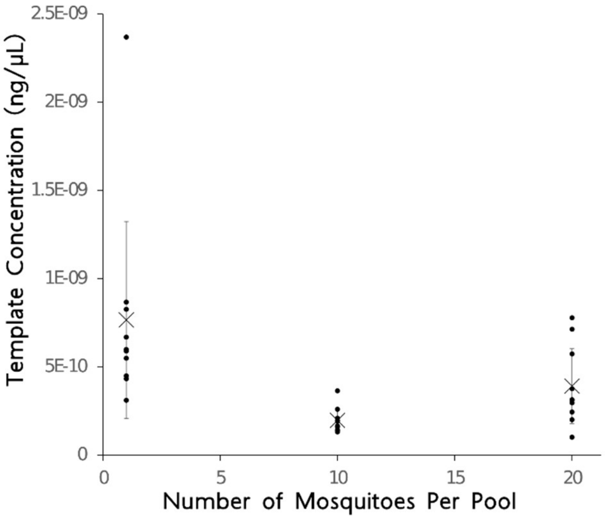

Figure S8. Barcode Detection Sensitivity. The recovered barcode amount (y-axis) plotted as a function of mosquito pool size (x-axis) demonstrates barcode DNA remains quantifiable at pools up to 20, proving detection sensitivity remains preserved despite greater pool sizes.

To determine the sensitivity of DNA detection as it relates to mosquito pool size, one crystal-fed mosquito was placed into pools of non-crystal-fed mosquitoes at increasing ratios. For the initial experiment, pool sizes of 1, 10 and 20 mosquitoes were assessed using 10 replicates per treatment (fig. S8). Observed melt curve variations, corresponding to samples SR1-3 in fig. 4, may result from variation in sample homogenization, although larger quantities of mosquito genomic DNA may be a contributing factor. Regardless, these data demonstrate that detection sensitivity of a single barcoded mosquito would not be compromised by pool size, for pools of up to 20 mosquitoes.

References

1. Stothard P (2000) The Sequence Manipulation Suite: JavaScript programs for analyzing and formatting protein and DNA sequences. *Biotechniques* 28:1102-1104.
2. Goswami J, Davis MC, Andersen T, Alileche A, & Hampikian G (2013) Safeguarding forensic DNA reference samples with nullomer barcodes. *J Forensic Leg Med* 20(5):513-519.
3. Newville M, Stensitzki, Till, Allen, Daniel B., Ingargiola, Antonio (2014) LMFIT: Non-Linear Least-Square Minimization and Curve-Fitting for Python. *Zenodo*.
4. Hartje LF*, et al.* (2018) Characterizing the Cytocompatibility of Various Cross-Linking Chemistries for the Production of Biostable Large-Pore Protein Crystal Materials. *Acs Biomater Sci Eng* 4(3):826-831.
