# Supplementary material for: Mosquito tagging using DNA-barcoded nanoporous protein microcrystals
Source: PNAS Nexus. 2022 Sep 12;1(4):pgac190. doi: 10.1093/pnasnexus/pgac190 (PMC9802479; doi:10.1093/pnasnexus/pgac190)
Supplement: pgac190_Supplemental_Files [file pgac190_supplemental_files.zip › PNASNEXUS-PNASNEXUS-2022-00358-T-s02.pdf]

Supplemental Information for
Mosquito Tagging Using DNA-Barcoded Nanoporous Protein Microcrystals

Authors

Julius D. Stuart[a,1], Daniel A. Hartman[b,c,1], Lyndsey I. Gray[b], Alec A. Jones[d], Natalie R. Wickenkamp[b], Christine Hirt[b,e], Aya Safira[d,f], April R. Regas[g], Therese M. Kondash[h,i], Margaret L. Yates[j], Sergei Driga[k], Christopher D. Snow[a,d,j,k], Rebekah C. Kading[b]

Author Affiliations

[a]Department of Chemistry, Colorado State University, Fort Collins, CO 80523; [b]Department of Microbiology, Immunology, and Pathology, Colorado State University, Fort Collins, CO 80523; [c]Department of Entomology, Cornell University, Ithaca NY 14853 (current);[d]School of Biomedical Engineering, Colorado State University, Fort Collins, CO 80523;[e]Invitae, Longmont, CO 80503 (current);[f]Just-Evotec Biologics, Seattle WA 98109 (current);[g]College of Veterinary Medicine and Biological Sciences, Colorado State University, Fort Collins, CO 80523;[h]Department of Environmental Health and Radiological Sciences, Colorado State University, Fort Collins, CO 80523; [i]H3 Environmental, Albuquerque, NM 87109 (current);[j]Department of Biochemistry and Molecular Biology, Colorado State University, Fort Collins, CO 80523; [k]Department of Chemical and Biological Engineering, Colorado State University, Fort Collins, CO 80523

Corresponding Author

Rebekah C. Kading
(970) 491-7833
Rebekah.Kading@colostate.edu
176 CVID
Colorado State University
Fort Collins, CO 80523

[1]J.D.S. and D.A.H. contributed equally to this work.

This PDF file includes:

Figures S9-12
Extended Materials and Methods

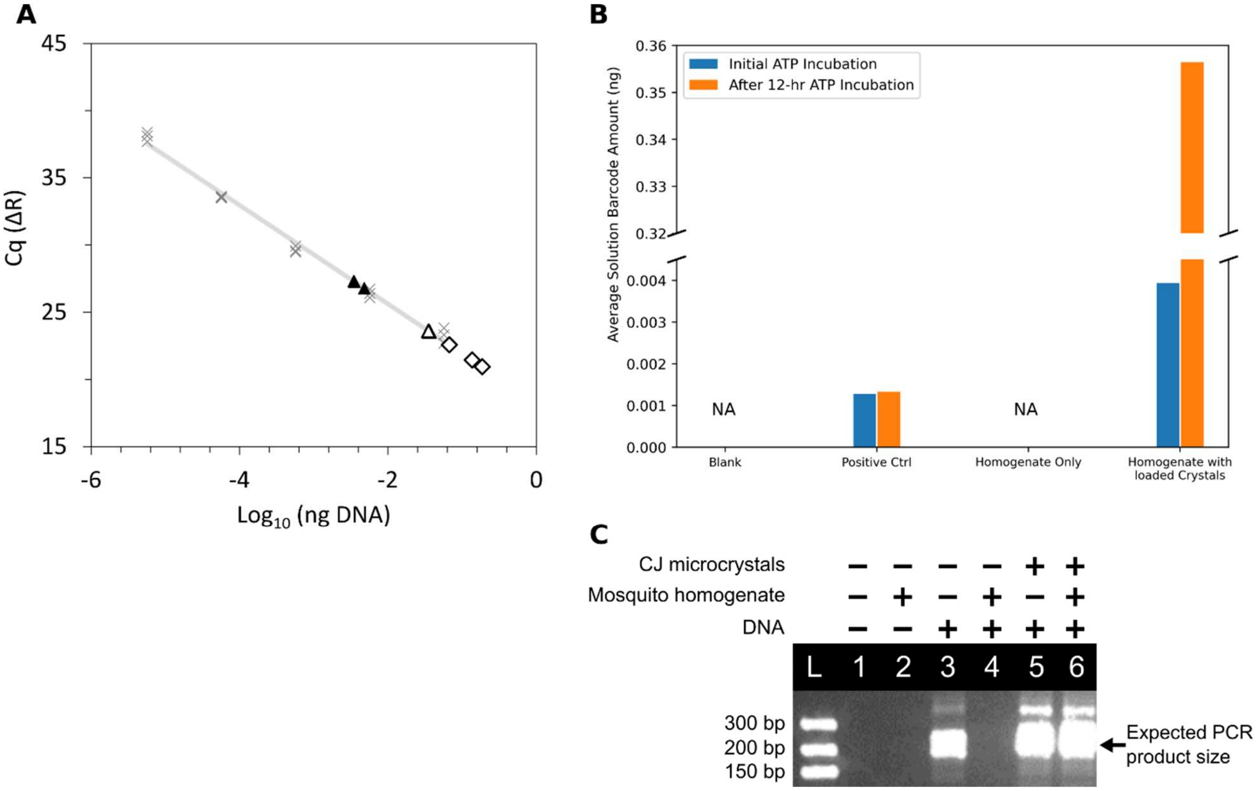

Figure S9. qPCR DNA Barcode Recovery Following Mosquito Homogenate Incubation and Crystal Protection. A) Standards plotted (x) with linear fit (gray line, R2 = 0.99). Triplicate measurements of the initial mosquito homogenate and crystal mixture demonstrated detectable barcode DNA in solution (black triangles). Incubation (12hr) with 20 mM ATP resulted in elevated barcode recovery (empty diamonds), exceeding the standard curve. A 10-fold dilution of the crystal/DNA/homogenate/ATP replicates brought the solution DNA concentration within a quantifiable range (empty triangles). **B)** Comparison of solution DNA Barcode amount upon initial ATP addition and after 12-hr incubation. No Cq values were detected for either the TE buffer blank or the homogenate only negative control. The solution DNA amount for the positive control (125mer in solution) remains virtually unchanged throughout the incubation period. Notably, the solution DNA amount for the crystal/homogenate mixture increases by over 2-orders of magnitude following ATP incubation. No Cq value was detected when using the 20 mM ATP as an additional negative control template (Data not shown). **C)** Barcode in solution was not detected following incubation with mosquito homogenate (lane 4) but was present in the sample that contained DNA-loaded microcrystals (lane 6) suggesting microcrystals protect DNA barcodes from degradation. Barcode detection was absent in the negative control samples, nuclease-free water (lane 1) and mosquito homogenate (lane 2). The expected PCR product was observed for both positive control samples, 200mer in solution (lane 3) and 200mer loaded in microcrystals (lane 5).

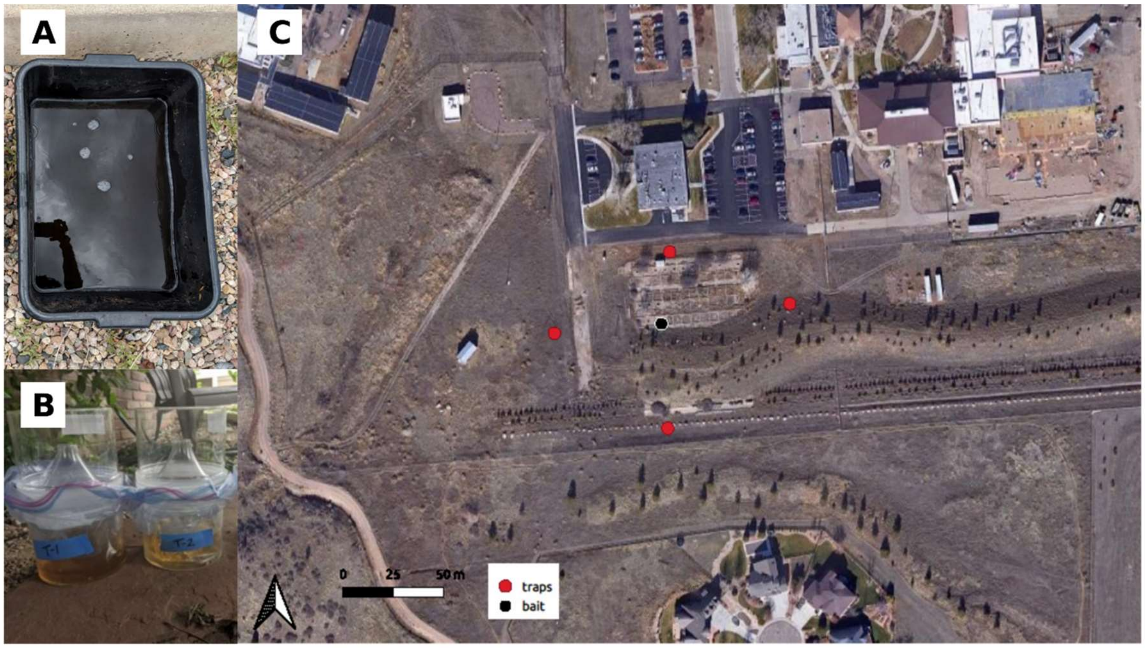

Figure S10. Pilot field test on the CSU Foothills Campus during the summer of 2020. **A)** Standardized concentrations of microcrystals were deployed into 18Q Sterilite wash basins, which also served to contain microcrystals within the environment. **B)** Mosquito rearing containers into which representative fourth instar larvae and pupae were picked from the wash basins and reared to adult mosquitoes in the insectary and assayed for the presence of barcode. **C)** Location of the pilot study on the CSU Foothills Campus. Red circles denote the locations of CDC light traps. Black circle denotes the location of the wash basin spiked with barcoded microcrystals. Scale bar denotes 50 meters. Microcrystal field deployments have been approved by the CSU Institutional Biosafety Committee (19-032B) which includes a local environmental risk assessment.

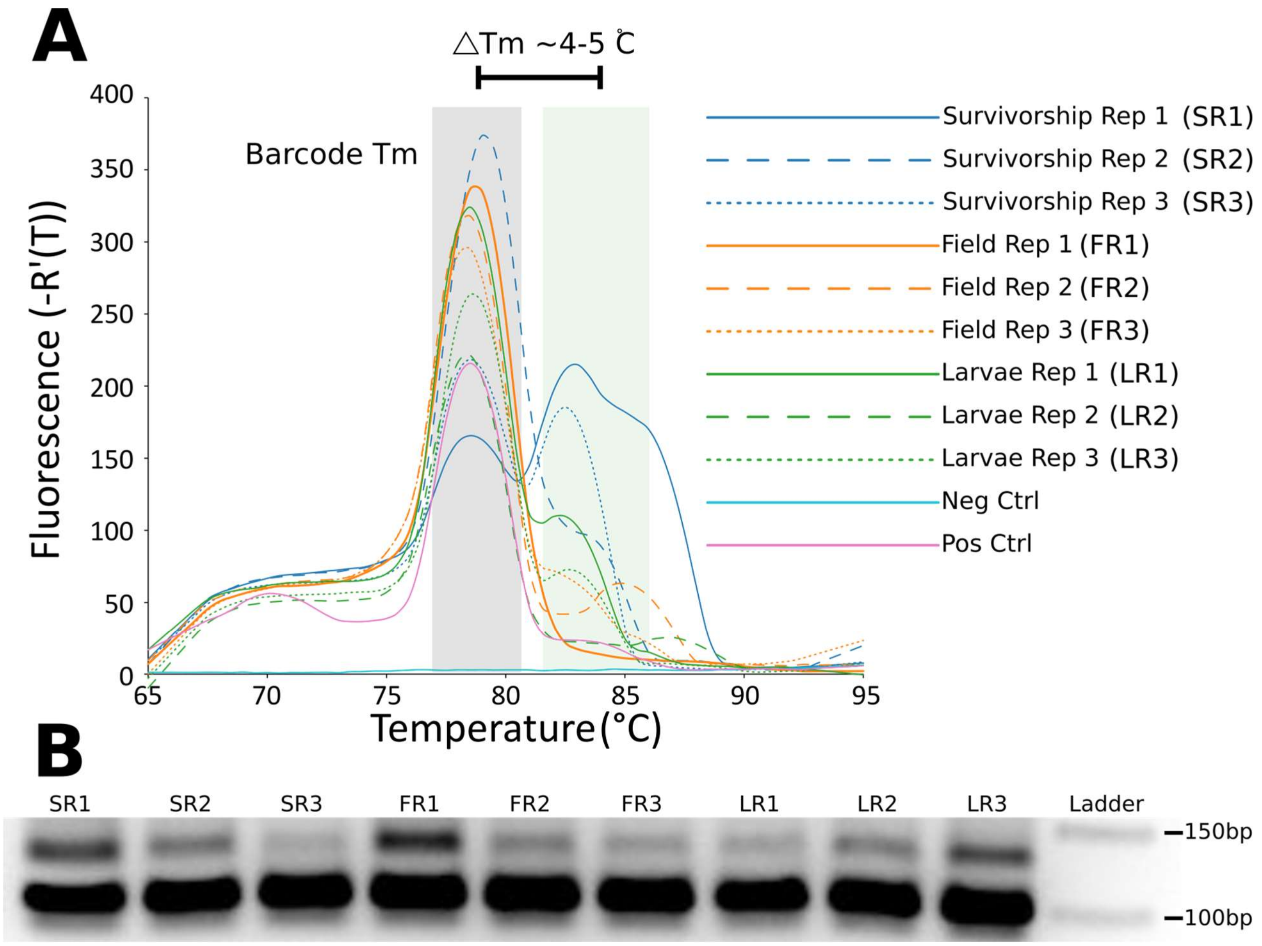

Figure S11. Barcode Detection for All Replicates. (A) qPCR melt curves for the three replicates from survivorship (SR1-3), field (FR1-3), and larvae (LR1-3) studies. **(B)** Gel electrophoresis results of all samples shown in (A) displaying the target 84-mer barcode band in addition to a fainter slightly higher band.

Figure S12. Field Trial qPCR Melt Curve Analysis (1/2). Representative melt curves from field collected mosquito pools (F) or laboratory-reared field collected mosquito larvae (L) followed by replicate number. As with survivorship samples, melt curves containing a peak at ~78.5 °C with a height at least 50% greater than the neighboring peak at ~ 74 °C using the python package LMFIT(3) were scored as positive. Left plots display raw qPCR melt curve data overlaid by the obtained fit along with the corresponding residuals. Right plots display raw qPCR data overlaid with the set of obtained gaussian peaks from computational analysis. The purple peak represents the detected DNA barcode. Analysis results of all samples are found on Zenodo (DOI: 10.5281/zenodo.6834837).

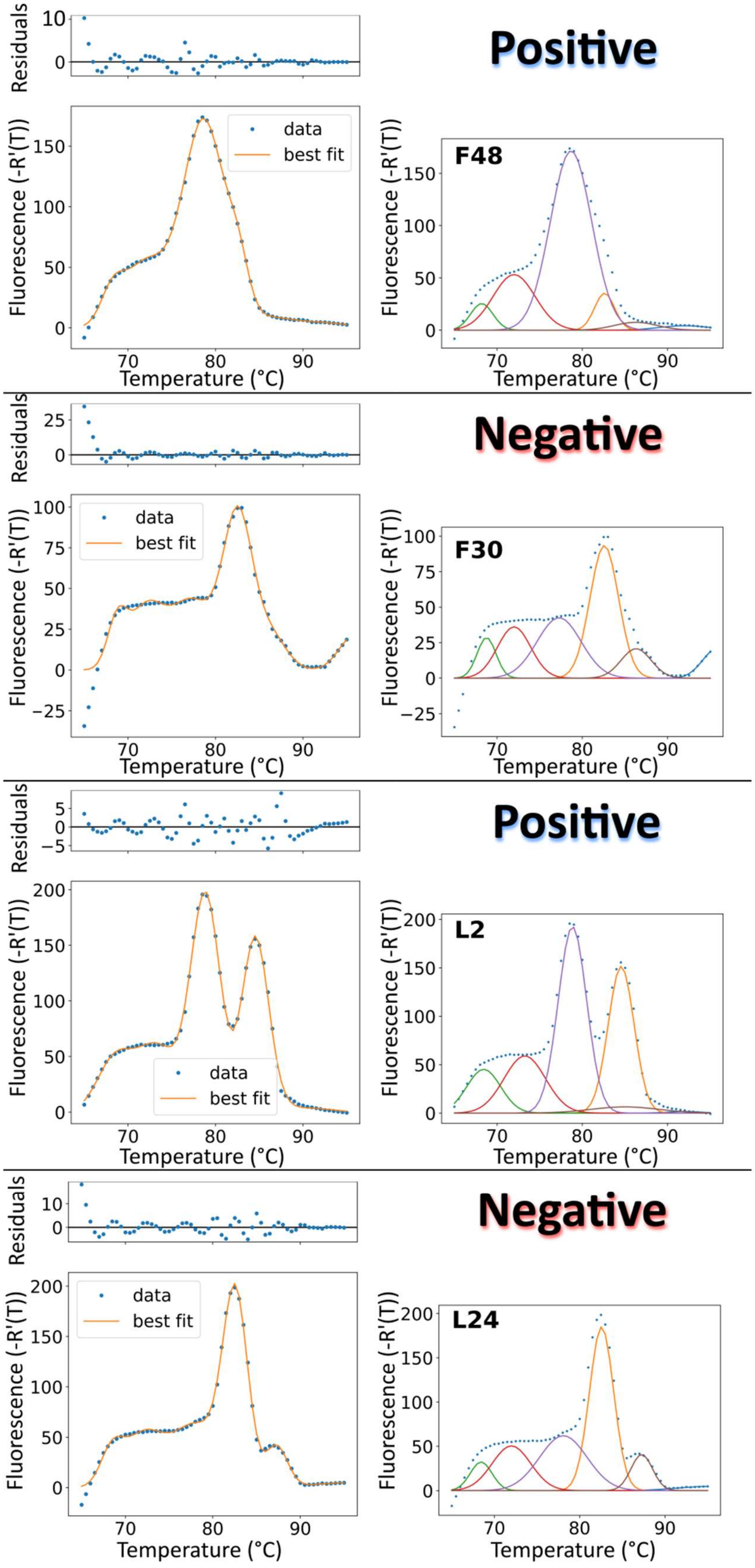

Figure S12. Field Trial qPCR Melt Curve Analysis (2/2). (A) Melt curves for a positive control (top) and negative control (bottom) overlaid with LMFIT(3) results. **(B)** Histogram of fitted barcode peak centers displaying a wider distribution of centers compared to survivorship samples.

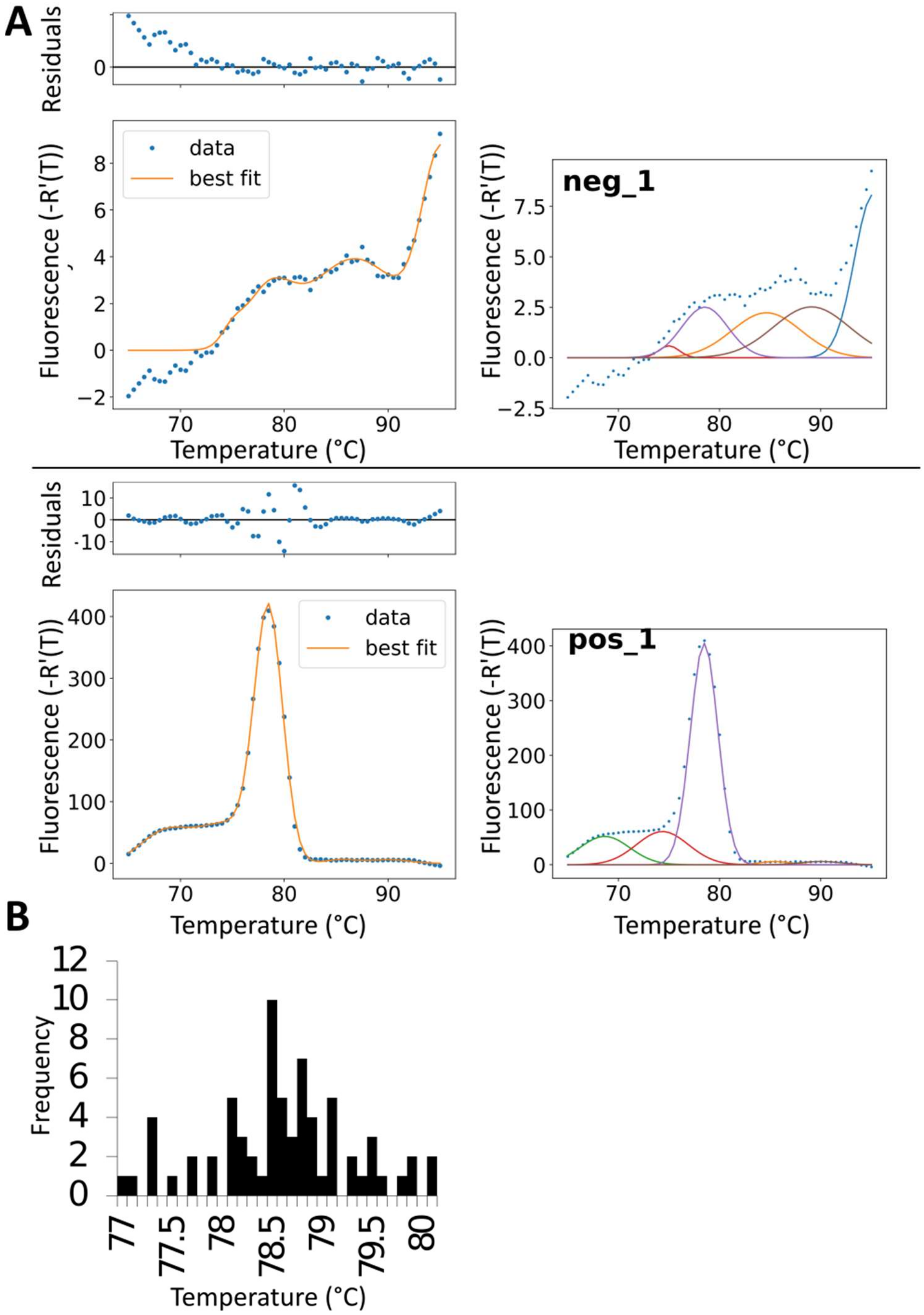

Extended Materials and Methods

Barcode Recovery from Homogenized Mosquitoes

Mosquitoes were homogenized in mosquito diluent (77.9% DMEM, 20% FBS, 1% penicillin/streptomycin, 1% amphotericin B, 0.1% gentamycin) using a bead beater (Retsch MM400) set at 24hz for 1 minute. ATP was added for a final concentration of 2mM prior to incubation. Sample homogenate was set on an orbital shaker at room temperature overnight for a minimum of 8h prior to extraction. Extraction was performed with a MaxMAX Cell-Free DNA extraction kit, using a modified protocol in a 96-well plate format on a Kingfisher Flex extraction platform (Thermo Fisher). qPCR was performed using PowerUP SYBR Green Master Mix (Thermo Fisher), with the primers, cycling conditions, and standard quantification described for in vivo qPCR barcode recovery.

Survival Analysis of Mosquitoes Reared on Crystals

Second instar *Culex tarsalis* mosquito larvae were reared in mosquito breeders (Bioquip), with 200 larvae in each container. The non-crystal fed control container received 200 µL of liver powder solution daily, and the crystal-reared treatment group received 200 µL of liver powder plus 25 µL of barcode-loaded crystal solution. Pupae were picked and placed individually in emergence containers. Upon emergence, mosquitoes received sugar cubes and water *ad libitum* and survivorship was followed until death. The following parameters were monitored: time to pupation, time to emergence, and time to death. Survival time was defined as the number of days from emergence to death. Mosquitoes found dead were frozen for barcode detection and determination of crystal persistence over time. Survival analysis was performed using a Bayesian survival model, with survival time following a Weibull distribution, and the mean of this distribution varying by group (crystal+liver powder vs. liver powder only) according to a linear regression function. Parameters were estimated by Markov Chain Monte Carlo using STAN. The regression coefficient for the 'group' factor did not differ significantly from zero, indicating no significant effect of crystal ingestion on adult mosquito survival. Plotting the posterior predictions show overlap between 95% credible intervals over the entire time course (fig. S4).

Barcode detection sensitivity

Culex tarsalis mosquito larvae were raised on a diet of CJ crystals loaded with DNA mixed into liver powder as previously described. Adult female mosquitoes were separated and frozen at -80°C. Non-crystal fed adult female mosquitoes were also separated and frozen. One crystal-fed mosquito was placed in samples of increasing numbers of non-crystal fed mosquitoes for totals of 1, 10, and 20 mosquitoes per pool, to represent the common pool sizes used for pathogen surveillance programs. Negative control groups consisted of the sample total number of mosquitoes (1, 10, 20) but without a crystal-fed mosquito. Mosquito pools were homogenized by adding 1mL of mosquito diluent (DMEM with 20% FBS, 50 ug/mL penicillin/streptomycin, 50 ug/ml gentamicin, 2.5 µl/ml fungizone), two glass Coliroller beads (Novagen), and 75µl of 100 mM ATP, and homogenizing for 3 minutes at 24 Hz using a Retsch Mixer Mill (Retsch). Barcodes were recovered from mosquitoes as described above.

in vitro qPCR validation

To prepare crystals for qPCR, 4 CJ crystals (~200 µm diameter) per replicate were immersed in 10 µL of approximately 50 ng/µL of a 125 base pair double-stranded DNA oligonucleotide (125mer, 5’-TAGGCGACTCGACGGTCTTACGCGTTACGTATGATATGCATCACCACCATC ACCAATAACCAACACCTAAATTTAACATCCGAGAATTATGGAGCACGCTAGCGTACGCTACGGTCCTAACGCGC-3’) and sealed in a glass well plate for approximately 12 hours, followed by washing with TE buffer as described for DNA loading to remove unbound 125mer. Following washing, 5 µL of solution was

removed from the DNA-loaded crystal mixture, followed by addition of 5 µL filtered mosquito homogenate and incubated for approximately 12 hours. Following incubation, 10 µL of 40 mM ATP were added to the crystal/DNA/homogenate mixture and an aliquot of the solution is stored. The 125mer loaded crystal/homogenate/ATP solution was sealed in a glass well plate for approximately 12 hours. Following the 12hr incubation, an additional aliquot of the solution is stored to compare solution 125mer concentration pre and post ATP incubation via qPCR with the following primers [31]: fwd 5'-TAGGCGACTCGACGGTCT TACGCGTTACGT-3', rev 5'- GCGCGTTAGGACCGTA GCGTACGCTAGCGT-3'. Standards used in qPCR consisted of serial dilutions of the pcr amplified 125mer template in nuclease free water. Starting with an initial 125mer template concentration of approximately 57 ng/µL, the following serial dilutions were made: 10^{-3}, 10^{-4}, 10^{-5}, 10^{-6}, and 10^{-7}. The quantitative amplification was performed per the manufacturer's instructions (Luna® Universal qPCR Master Mix). Reaction conditions were: 1 cycle of 95 °C for 3:00 min and 40 cycles of 95 °C for 15 seconds followed by 72 °C for 30 seconds. Melt curve was obtained by 1 cycle of 95 °C for 30 seconds, 1 cycle of 65 °C for 30 seconds and 1 cycle of 95 °C for 30 seconds. TE buffer and filtered mosquito homogenate were separately used as the templates for negative controls. The positive control was 125mer in solution.

in vivo Environmental Persistence qPCR

A master mix was prepared using 2X qPCR mix (Agilent #600882), 125mer revised forward primer (10 µM), 125mer reverse primer (10 µM) and nuclease water. Master mix (14 µL) was combined with 6 µL of unknown sample, template DNA or water. Standard curves were prepared using 4 to 5 100-fold serial dilutions of known concentration of barcode DNA. All reactions were performed in duplicate under the following cycling conditions: 1 cycle of 95 °C for 3:00 min and 50 cycles of 95 °C for 5 s, 60 °C for 10 s. Melt curve analysis was performed by spanning 65 °C to 95 °C, + 0.5 °C/cycle with 5 s/cycle. Initial concentrations were extrapolated from standard curves for each qPCR run. These quantities were combined into a single Excel spreadsheet. Single-factor ANOVA was used to assess statistical significance, before and after omitting samples with a Cq standard deviation ≥ 1. With inclusion of the omitted data, this result remains statistically significant ($p = 0.029$, $n = 42$ for loaded crystals and $n = 45$ for naked DNA).

Porous Crystal Production

Briefly, protein possessing a C-terminal hexahistidine tag was cloned into pSB3 expression vector. Protein expression was performed in BL21(DE3) *Escherichia coli* cells using Terrific broth with 0.4 mM IPTG induction at 25 °C for 16 hours. Cells were spun down, resuspended in lysis buffer (50 mM HEPES, 500 mM NaCl, 25 mM Imidazole, 10 % glycerol, pH 7.4), and sonicated. Cell lysate was purified using immobilized metal affinity chromatography (IMAC) containing HisPur Ni-NTA resin (Thermo Fisher Scientific) and dialyzed into ammonium sulfate storage buffer (10 mM HEPES, 500 mM $NH_4(SO_4)_2$, 10% glycerol, pH 7.4) overnight at 4 °C. Purified protein was concentrated to 15 mg/mL by using Amicon Ultra-15 Centrifugal Filters (MWCO 10K, Millipore Sigma), aliquoted and stored at -30 °C. Crystals were grown overnight with sitting drop vapor diffusion (for single-crystal confocal imaging) or batch crystallization (for mosquito feeding) in 3.3 – 3.55M ammonium sulfate, 0.1 M Bis-Tris, pH 6.5 at 20 °C. Crystal cross-linking was performed as described previously(4). Crystals were transferred to 4.2 M trimethylamine N-oxide (TMAO), pH 7.4 for 1 hour to remove excess protein in solution. Crystals were then placed in fresh 4.2M TMAO containing 50 mM imidazole and 40 mg/mL 1-ethyl-3-(3-dimethylaminopropyl) carbodiimide hydrochloride (EDC) for 2 hours. Crystals were then placed in quench solution (50 mM borate, pH 10) for 1 hour, followed by washing and storage in 4.2 M TMAO, pH 7.4.

Crystal Fluorophore Labeling

Following crystallization, crystals were loop-transferred into a wash solution comprised of 90% mTacsimate (1.83 M malonic acid, 0.25 M sodium citrate, 0.12 M succinic acid, 0.3 M D-L malic acid, 0.4 M acetic acid, 0.5 M sodium formate, 0.16 M sodium tartrate, pH 6.5) and 10% glycerol for 1 hour. This is an altered recipe based on Tacsimate (Hampton Research) that does not contain primary amines that could interfere with crosslinking. Crystals were crosslinked in fresh wash solution containing 1% glyoxal and 250 mM borane dimethylamine complex (DMAB) for 2 hours. Crystals were quenched and labeled with Texas Red dye by transferring to quench solution containing 0.25 M carbohydrazide, 0.25 mM Texas Red-X (ThermoFisher), and 100 mM DMAB in phosphate buffered saline (137 mM sodium chloride, 2.7 mM potassium chloride, 10 mM sodium phosphate dibasic, 1.8 mM potassium phosphate dibasic), pH 7.5 for 1 hour. Following crosslinking and labeling, crystals were washed and stored in 4.2 M TMAO.

References

1. Stothard P (2000) The Sequence Manipulation Suite: JavaScript programs for analyzing and formatting protein and DNA sequences. *Biotechniques* 28:1102-1104.
2. Goswami J, Davis MC, Andersen T, Alileche A, & Hampikian G (2013) Safeguarding forensic DNA reference samples with nullomer barcodes. *J Forensic Leg Med* 20(5):513-519.
3. Newville M, Stensitzki, Till, Allen, Daniel B., Ingargiola, Antonio (2014) LMFIT: Non-Linear Least-Square Minimization and Curve-Fitting for Python. *Zenodo*.
4. Hartje LF*, et al.* (2018) Characterizing the Cytocompatibility of Various Cross-Linking Chemistries for the Production of Biostable Large-Pore Protein Crystal Materials. *Acs Biomater Sci Eng* 4(3):826-831.
